# A regional assessment of white-tailed deer effects on plant invasion

**DOI:** 10.1093/aobpla/plx047

**Published:** 2017-12-07

**Authors:** Kristine M Averill, David A Mortensen, Erica A H Smithwick, Susan Kalisz, William J McShea, Norman A Bourg, John D Parker, Alejandro A Royo, Marc D Abrams, David K Apsley, Bernd Blossey, Douglas H Boucher, Kai L Caraher, Antonio DiTommaso, Sarah E Johnson, Robert Masson, Victoria A Nuzzo

**Affiliations:** Ecology Intercollege Graduate Degree Program, The Pennsylvania State University, University Park, PA, USA; Department of Plant Sciences, The Pennsylvania State University, University Park, PA, USA; Department of Geography, The Pennsylvania State University, University Park, PA, USA; Department of Ecology and Evolutionary Biology, University of Tennessee, Knoxville, TN, USA; Smithsonian Conservation Biology Institute, Front Royal, VA, USA; Smithsonian Environmental Research Center, Edgewater, MD, USA; United States Department of Agriculture Forest Service, Northern Research Station, Irvine, PA, USA; Department of Ecosystem Science and Management, The Pennsylvania State University, University Park, PA, USA; Department of Extension, The Ohio State University, Jackson, OH, USA; Department of Natural Resources, Cornell University, Ithaca, NY, USA; Department of Biology, Hood College, Frederick, MD, USA; Soil and Crop Sciences Section, Cornell University, Ithaca, NY, USA; National Park Service, Morristown National Historical Park, Morristown, NJ, USA; Natural Area Consultants, Richford, NY, USA

**Keywords:** Biological invasions, exotic plants, herbivory, introduced plants, *Odocoileus virginianus*, palatability, plant invasion, regional pooled analysis

## Abstract

Herbivores can profoundly influence plant species assembly, including plant invasion, and resulting community composition. Population increases of native herbivores, e.g. white-tailed deer (*Odocoileus virginianus*), combined with burgeoning plant invasions raise concerns for native plant diversity and forest regeneration. While individual researchers typically test for the impact of deer on plant invasion at a few sites, the overarching influence of deer on plant invasion across regional scales is unclear. We tested the effects of deer on the abundance and diversity of introduced and native herbaceous and woody plants across 23 white-tailed deer research sites distributed across the east-central and north-eastern USA and representing a wide range of deer densities and invasive plant abundance and identity. Deer access/exclusion or deer population density did not affect introduced plant richness or community-level abundance. Native and total plant species richness, abundance (cover and stem density) and Shannon diversity were lower in deer-access vs. deer-exclusion plots. Among deer-access plots, native species richness, native and total cover, and Shannon diversity (cover) declined as deer density increased. Deer access increased the proportion of introduced species cover (but not of species richness or stem density). As deer density increased, the proportion of introduced species richness, cover and stem density all increased. Because absolute abundance of introduced plants was unaffected by deer, the increase in proportion of introduced plant abundance is likely an indirect effect of deer reducing native cover. Indicator species analysis revealed that deer access favoured three introduced plant species, including *Alliaria petiolata* and *Microstegium vimineum*, as well as four native plant species. In contrast, deer exclusion favoured three introduced plant species, including *Lonicera japonica* and *Rosa multiflora*, and 15 native plant species. Overall, native deer reduced community diversity, lowering native plant richness and abundance, and benefited certain invasive plants, suggesting pervasive impacts of this keystone herbivore on plant community composition and ecosystem services in native forests across broad swathes of the eastern USA.

## Teaser

Analyses deriving insights from 23 research sites across the northeastern and east central US reveals that white-tailed deer lower native plant diversity and increase the fraction of the plant community made up of non-native species. Particularly, deer increase the abundance of the invasive plants garlic mustard and Japanese stiltgrass. Deer are known to avoid eating these species in favour of more palatable ones, thereby indirectly facilitating the success of the non-native invasives. Managing deer abundance has implications for forest plant communities. By maintaining lower deer densities, native plants likely will be better sustained and invasions of unpalatable non-native plants limited.

## Introduction

Modern plant communities are anthropogenically altered (Hannah *et al.* 1994). Habitat loss and forest fragmentation have contributed to acute reductions in biodiversity, species homogenization, and concomitant proliferation of invasive species and some large herbivores (McKinney and Lockwood 1999; Rooney *et al.* 2004; Alroy 2008). Because large mammalian herbivores can play a prominent role in determining plant community composition (Harper 1977; Crawley 1997; Russell *et al.* 2001; Côté *et al.* 2004), understanding their effects on plant species and communities, including plant invasions, is critical for conserving biodiversity.

Large herbivores affect plant communities directly via tissue loss and plant mortality, indirectly through non-consumptive effects including trampling (Persson *et al.* 2000; Heckel *et al.* 2010), accelerating nutrient cycling (Hobbs 1996; Rooney and Waller 2003) and by dispersing plant propagules (Vellend 2002; Myers *et al.* 2004; Bartuszevige and Endress 2008; Williams *et al.* 2008; Castellano and Gorchov 2013). Perhaps the most pervasive effect of large mammals on plant communities, however, is their indirect impact of altering interspecific plant competition through selective herbivory and plant response to herbivory (Holt 1977; Bowers 1993; Crawley 1997; Augustine and McNaughton 1998), with large impacts on community assembly and succession (Drake 1990; Hobbs 1996). For example, herbivores can alter successional trajectories when they preferentially consume early or late successional plant species (Hobbs 1996; Crawley 1997; Côté *et al.* 2004; DiTommaso *et al.* 2014; Forsyth *et al.* 2015). Consumption of palatable species can cause unpalatable species to gain an apparent competitive advantage and potentially become dominant or invasive (Leopold *et al.* 1947; Holt 1977; Augustine and McNaughton 1998; Horsley *et al.* 2003; Côté *et al.* 2004; Vavra *et al.* 2007). For example, pastures and rangeland can become infested with *Carduus*, *Centaurea* and *Cirsium* spp., among others, when grazers consume more palatable species (DiTomaso 2000). Selective herbivory can result in woody plant invasion in savannas, i.e. encroachment, which occurs as grazers reduce herbaceous species, indirectly facilitating establishment of unpalatable woody vegetation (Asner *et al.* 2004), but the more common result is a reduction of palatable woody plants, which slows succession from field to forest (DiTommaso *et al.* 2014; Habeck and Schultz 2015). The selective browsing of cervids (e.g. deer, moose, elk) is considered one of the main determinants of forest understory plant species composition and structure (Alverson *et al.* 1988; Côté *et al.* 2004; Abrams 2013). Herbivore-mediated shifts in plant communities can limit native plant regeneration, alter the abundance of small mammals, birds and insects, lower ecological stability (e.g. erosion and flood protection), disrupt ecosystem functioning, induce alternative stable states, reduce the economic value of habitats (reviewed in Côté *et al.* 2004) and trigger or facilitate plant invasions (Stromayer and Warren 1997; Vavra *et al.* 2007).

In North America, many large native herbivores, including bison (*Bison bison*), caribou (*Rangifer tarandus*), Dall’s sheep (*Ovis dalli*), elk (*Cervus elaphus*), moose (*Alces alces*) and pronghorn (*Antilocapra americana*), have experienced severe range contractions during the past 200 years (Laliberte and Ripple 2004). However, the range and abundance of native white-tailed deer (*Odocoileus virginianus*; hereafter referred to as deer) increased steadily following steep population declines in the late 1800s (McCabe and McCabe 1997; Laliberte and Ripple 2004). Low predator populations (Laliberte and Ripple 2004) and game laws that restricted hunting, in addition to increasing agricultural, silvicultural and early successional habitat, enhanced deer habitat within the past century, resulting in high deer populations (Alverson *et al.* 1988; McShea *et al.* 1997; Waller and Alverson 1997; Côté *et al.* 2004). Today, deer are the dominant wild ruminant herbivore in east-central and north-eastern USA and, because of their high abundance, are a serious ecological and management concern (McShea *et al.* 1997; Rooney 2001; McShea 2012). While deer at low abundances can increase floristic diversity (Royo *et al.* 2010a; Cook-Patton *et al.* 2014), abundant deer limit diversity and promote floral homogeneity (Rooney *et al.* 2004; Wiegmann and Waller 2006). At chronically high densities, deer change plant community structure and composition enough to be considered ‘ecosystem engineers’ or ‘keystone herbivores’ (Alverson *et al.* 1988; Waller and Alverson 1997; Côté *et al.* 2004). In many areas, deer population densities greatly exceed ecosystem carrying capacity (Rooney and Waller 2003), causing long-lasting and potentially irreversible legacy effects (Royo *et al.* 2010b; Nuttle *et al.* 2011).

As deer abundance increased during the past century, so did abundance of introduced plants, resulting in often concurrent ecological impacts. Human transport facilitates movement of many species outside their native ranges and, consequently, non-native species are now prominent components of present-day communities (Lockwood *et al.* 2013). Introduced plant species pose a growing threat to native plant communities, as their presence is associated with altered diversity, community structure and ecosystem function (MacDougall and Turkington 2005; Ehrenfeld 2010; Vilà *et al.* 2011; Beasley and McCarthy 2011). The fact that populations of deer and introduced plants have expanded concurrently suggests that deer abundance might be linked to introduced plant invasions (Augustine and McNaughton 1998; Vavra *et al.* 2007). However, data are lacking on regional effects of deer on native plant communities and plant invasion (Maron and Vilà 2001; Russell *et al.* 2001; Mosbacher and Williams 2009).

Throughout the past century, numerous experiments using fenced (deer-exclusion) and unfenced (deer-access) plots gauged deer impacts on forest plant communities (e.g. see McShea *et al.* 1997; Côté *et al.* 2004; Abrams and Johnson 2012; Habeck and Schultz 2015). Use of paired plots affords valuable insight into effects of large herbivores on floristic composition and on native and introduced plants, yet site-level studies assessing the degree to which deer influence introduced plants have yielded equivocal results. Several paired-plot experiments report deer facilitate certain invasive plants (Knight *et al.* 2009; Eschtruth and Battles 2009b; Beasley and McCarthy 2011; Kalisz *et al.* 2014; Dávalos *et al.* 2015b), others report deer mitigate invasions of different species (Rossell *et al.* 2007; Shelton *et al.* 2014) and others find no effect (Bowers 1993; Levine *et al.* 2012; DiTommaso *et al.* 2014) or mixed effects (Cadenasso *et al.* 2002; Webster *et al.* 2005; Knapp *et al.* 2008; Shen *et al.* 2016). Site-level investigations can provide practical insights about local species and conditions, but cannot be extrapolated to regional assessments about deer herbivory and plant invasion. A regional assessment requires data on a range of plant community types across a range of deer densities. Spatially broad investigations can bolster generalizations and forecasts made about ecological processes (Clark *et al.* 1999, 2001), such as community assembly and plant invasion (Gill and Beardall 2001; Shen *et al.* 2016).

Here, we present results of a multisite, regional assessment of white-tailed deer effects on composition, richness and abundance of introduced and native plants in east-central and north-eastern USA. We pool data from 23, paired-plot deer access/exclusion experiments spanning a broad range of invasive plant abundance and deer densities. We predicted that deer access would (i) alter floristic composition and reduce floristic diversity and (ii) increase richness and abundance of introduced plant species and decrease richness and abundance of native plant species.

## Methods

### Data description

**Figure 1.**
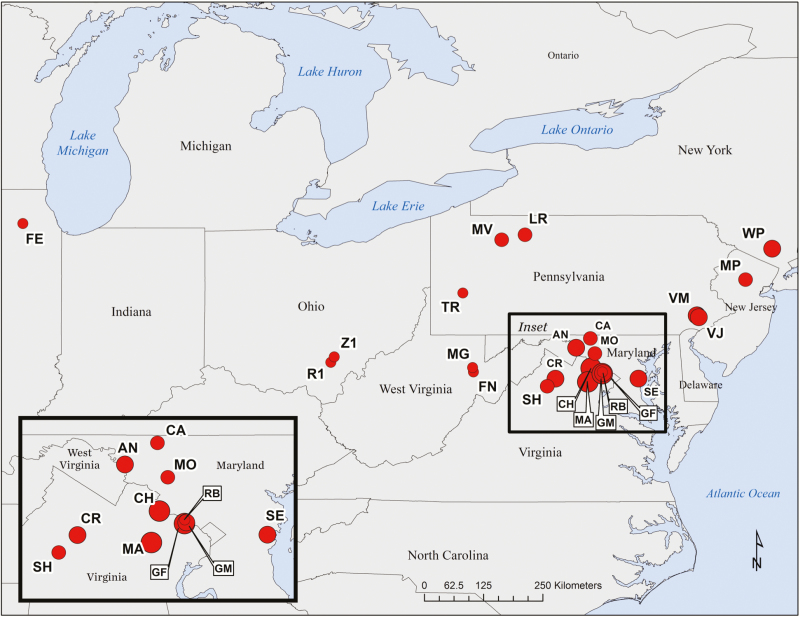
Locations of 23 deer research sites in east-central and north-eastern USA included in pooled analyses. Symbol size indicates sampling intensity across sites, which are labelled with two-letter codes (see Table 1 for additional site information).

We compiled data sets in which herbaceous and woody floristic composition and abundance were quantified in replicated deer-exclusion and deer-access plot experiments across 23 sites, resulting in 446 experimental units (223 plot pairs) (Table 1). We acquired data sets by directly contacting investigators of previously published (15 sites) and unpublished data (6 sites) and collecting additional data from established plots (2 sites, Long Run and Marienville) (Table 1). Sites were located in temperate deciduous or mixed deciduous forests across east-central and north-eastern USA (Table 1; Fig. 1). Sites were initially established to answer a range of research questions, not solely the effects of deer on introduced plants (Table 1). Overstory species typically included oak (*Quercus* spp.), maple (*Acer* spp.), beech (*Fagus grandifolia*), tulip-poplar (*Liriodendron tulipifera*) and black cherry (*Prunus serotina*) (Table 1). Deer density estimates varied across sites from 4 to 107 deer km^−2^ (Table 1; for estimation methods used, **see Supporting Information—Table S1**). The timing and duration of deer exclusion varied across experiments (Table 1). Six sites were established in the late 1980s/early 1990s, and the remaining 17 sites in the 2000s. At 15 sites, deer exclusion was imposed for 6 years or less, while at the other eight sites it ranged from 8 to 17 years. During the summer growing season, abundance data of herbaceous and woody species up to 2 m in height was recorded. Sampling intensity, plot area and replication varied across sites (Table 1). A recent meta-analysis showed no relationship between plot area and plant community responses to deer (Habeck and Schultz 2015). Fence heights used to exclude deer were a minimum of 2 m. Fence mesh size varied across experiments; therefore, deer may not have been the only mammalian herbivore excluded (e.g. see Bowers 1993).

**Table 1.** Descriptions of 23 experimental sites and data used in pooled analyses testing the effect of white-tailed deer on introduced and native plants in east-central and north-eastern USA. Floristic composition data were collected from deer-access (unfenced) and Table 1A deer-exclusion (fenced) plots.

Site (code)	US state	Latitude	Longitude	Dominant overstory species	Vegetation abundance measurement	Reference used for plant classification	Initial purpose/establishment of experiment	Estimated deer density^c^	Duration of deer exclusion	Years of study	# Plot pairs	Plot area^d^	Subplot area	Total area sampled/plot	Distance between paired plots	Fence height	Fence mesh size	Data source; notes
	Decimal degrees																	
Antietam National Battlefield (AN)	MD	39.4763	−77.7490	Maple, white ash, cherry	Density, cover classes^a^ or density classes in subplots; sapling density in main plot	Strausbaugh and Core (1978); Brown and Brown (1984); Gleason and Cronquist (1991)	Woody seedling establishment^b^	53	6	2003–09	12	25	1	4	<5	2.4	10 × 10	McShea and Bourg (2009)
Catoctin Mountain Park (CA)	MD	39.6561	−77.4786	Maple, tulip poplar	Density or cover classes	Newcomb (1977); USDA NRCS (2012)	Deer effects on plant composition in blow-down gaps created by hurricane Ivan	44	3	2005–08	7	25	None	25	5	3	10 × 20	Caraher (2009)
Chesapeake & Ohio Canal National Historical Park (CH)	MD	39.0882	−77.4619	Maple, white ash, cherry	Density, cover classes^a^ or density classes in subplots; sapling density in main plot	Strausbaugh and Core (1978); Brown and Brown (1984); Gleason and Cronquist (1991)	Woody seedling establishment^b^	54	6	2003–09	28	25	1	4	<5	2.4	10 × 10	McShea and Bourg (2009)
Smithsonian Conservation Biology Institute (CR)	VA	38.8885	−78.1434	Oak, beech	Density, cover classes^a^ or density classes	Strausbaugh and Core (1978); Brown and Brown (1984); Gleason and Cronquist (1991)	Deer and invasive plant interactions in upland forest	107	4	2005–09	14	16	1	4	50	2.4	5 × 5	Unpublished data, W. J. McShea and N. A. Bourg, SI Conservation Biology Institute
Smithsonian Environmental Research Center (SE)	MD	38.8908	−76.5646	Tulip poplar, sweet gum, beech	Per cent cover	Gleason and Cronquist (1991); botanists (see note)	Deer effects on plant composition (random site selection)	4	2	2009–11	16	100	1	5	3–10	2.3	50 × 50	Unpublished data, J. D. Parker, SI; species ID: pers. comm. with botanists at SI Museum of Natural History
Fermilab (FE)	IL	41.8423	−88.2631	Oak, ash, basswood	Cover classes	Swink and Wilhelm (1994)	Vegetation recovery after deer exclusion in two upland forests, one with historically rich flora	6	14	1992–2006	3	594	1	25	5	3	15 × 15	Unpublished data, V. Nuzzo, Natural Area Consultants; 90% deer herd cull in 1998
Fernow (FN)	WV	39.0167	−79.7000	Oak, maple, beech	Density and per cent cover	Gleason and Cronquist (1991); USDA NRCS (2012)	Disturbance and deer interactions	6	6	2000–06	4	400	1	5	>20	2	15 × 15 or 30	Royo *et al.* (2010a)
Gold Mine Tract of C&O Canal (GM)	MD	38.9931	−77.2392	Oak, beech	Density, cover classes^a^ or density classes	Strausbaugh and Core (1978); Brown and Brown (1984); Gleason and Cronquist (1991)	Deer and invasive plant interactions in upland forest	45	4	2005–09	10	16	1	4	50	2.4	5 × 5	McShea and Bourg (2008)
Great Falls Park (GF)	VA	38.9840	−77.2531	Oak, beech	Density, cover classes^a^ or density classes	Strausbaugh and Core (1978); Brown and Brown (1984); Gleason and Cronquist (1991)	Deer and invasive plant interactions in upland forest	26	4	2005–09	22	16	1	4	50	2.4	5 × 5	McShea and Bourg (2008)
Long Run (LR)	PA	41.6288	−78.7211	Black cherry, red maple	Per cent cover	Rhoads *et al.* (2007); USDA NRCS (2012); botanists (see note)	Deer and fern effects on woody seedling recruitment	5	11	2000–11	5	280	1	4	10–30	2	5 × 5	Unpublished data, K. M. Averill and D. A. Mortensen and A. A. Royo, USDA Forest Service; species ID: pers. comm. with botanists at Penn State
Manassas National Battlefield Park (MA)	VA	38.8266	−77.5279	Oak, hickory, VA pine, northern red cedar	Density and per cent cover	Strausbaugh and Core (1978); Brown and Brown (1984); Gleason and Cronquist (1991)	Woody seedling establishment^b^	63	9	2000–09	23	12	1	4	1	2	5 × 10	McShea *et al.* (2010)
Marienville (MV)	PA	41.5347	−79.1643	Black cherry, red maple	Per cent cover	Rhoads *et al.* (2007); USDA NRCS (2012); botanists (see note)	Deer and fern effects on woody seedling recruitment	5	11	2000–11	5	280	1	4	10–30	2	5 × 5	Unpublished data, K. M. Averill and D. A. Mortensen and A. A. Royo, USDA Forest Service; species ID: pers. comm. with botanists at Penn State
Monocacy National Battlefield (MO)	MD	39.3697	−77.3924	Dry oak, tulip poplar	Density or cover classes^a^ in subplots; sapling density in main plot	Strausbaugh and Core (1978); Brown and Brown (1984); Gleason and Cronquist (1991)	Woody seedling establishment^b^	77	6	2003–09	6	25	1	4	<5	2.4	10 × 10	McShea and Bourg (2009)
Monongahela (MG)	WV	39.1000	−79.7167	Oak, maple, beech	Density and per cent cover	Gleason and Cronquist (1991); USDA NRCS (2012)	Disturbance and deer interactions	6	6	2000–06	4	400	1	5	>20	2	15 × 15 or 30	Royo *et al.* (2010a)
Morristown National Historic Park (MP)	NJ	40.7760	−74.5301	Tulip poplar, white ash, oak, black locust	Cover classes	Newcomb (1977); Gleason and Cronquist (1991)	Plant composition and community structure	19	14–17	1987–2005	5	36	1	9	~9	3.7	11 × 15	Unpublished data, R. Masson, National Park Service
Raccoon Ecological Management Area (R1)	OH	39.1997	−82.4093	Oak, hickory	Cover classes	Gleason and Cronquist (1991)	Acorns and oak regeneration (stratified random sampling)	11	5	2001–06	3	400	1	12	<5	2.4	4.4 × 5	Unpublished data, T. Hutchinson and D. K. Apsley, USDA Forest Service
Riverbend Park (RB)	VA	39.0145	−77.2522	Oak, beech	Density, cover classes^a^, or density classes	Strausbaugh and Core (1978); Brown and Brown (1984); Gleason and Cronquist (1991)	Deer and invasive plant interactions in upland forest	26	3	2006–09	2	16	1	4	50	2.4	5 × 5	McShea and Bourg (2008)
Shenandoah National Park (SH)	VA	38.7438	−78.2992	Oak, hickory, pine	Density or density classes	Gleason and Cronquist (1991)	Acorn, rodent, bird interactions; deer and ecosystem interactions	10	4–6	1990–96	6	4 ha	1	18	>1 km	2.4	15 × 15	McShea and Rappole (2000); McShea (2000); plots paired regionally, each with 3 24 × 24 m plots
Trillium Trail (TR)	PA	40.5201	−79.9011	Oak, beech, maple, tulip poplar	Per cent cover	Gleason and Cronquist (1991)	Paired plots established to contain same native species with similar abiotic conditions	32	8	1994–2002	3	100	None	100	~60	2.5	6 × 6	Knight *et al.* (2009)
Valley Forge National Historical Park–Mt Joy (VJ)	PA	40.0940	−75.4543	Tulip poplar, dry oak	Cover classes or density	Gleason and Cronquist (1991)	Plant composition; largest contiguous park woodlands selected	84	17	1993–2010	15	9	4	4	20–36	2	5 × 10	Abrams and Johnson (2012); 2 m metal stake in centres of control plots
Valley Forge National Historical Park–Mt Misery (VM)	PA	40.0932	−75.4611	Dry oak	Cover classes or density	Gleason and Cronquist (1991)	Plant composition; largest contiguous park woodlands selected	84	17	1993–2010	15	9	4	4	20–36	2	5 × 10	Abrams and Johnson (2012); 2 m metal stake in centres of control plots
West Point (WP)	NY	41.3636	−74.0239	Oak, sugar maple	Cover classes	Rhoads *et al.* (2007); USDA NRCS (2012)	Multiple stressor effects including deer and invasive plants; upland forests selected, half with invasive plants and half with none, without knowledge of deer abundance	No estimate available	4	2008–12	12	900	1	10	5–50	2.4	5 × 5	Nuzzo *et al.*, this issue
Zaleski (Z1)	OH	39.3032	−82.3461	Oak, hickory	Cover classes	Gleason and Cronquist (1991)	Acorns and oak regeneration	11	5	2001–06	3	400	1	12	<5	2.4	4.4 × 5	Unpublished data, T. Hutchinson and D. K. Apsley, USDA Forest Service

We acknowledge that the paired-plot approach has limitations, including fence-line effects, fences providing artificial support for vines and concentrated perch areas for birds, and an unrealistic total absence of deer in fenced plots (Russell *et al.* 2001; White 2012). Deer also presumably exerted an influence prior to experimentation (Russell *et al.* 2001), leaving behind legacy effects even after culling (Royo *et al.* 2010b; Nuttle *et al.* 2011), such as altered seedbank composition (DiTommaso *et al.* 2014), which could limit vegetation response to deer exclusion. Beyond the scope of this work, drivers of invasion could vary between areas with deer access vs. deer exclusion. Despite these limitations, herbivore-exclusion experiments remain among the most straightforward of ways to test the effects of herbivores on plant invasions (e.g. Parker *et al.* 2006).

### Data set pooling

The pooling approach taken here has the benefit of increasing statistical power and reducing type II error rates (i.e. false negatives; Blettner *et al.* 1999). We processed the most recent floristic assessment from each experiment to analyse vegetation patterns at single points in time. However, we recognize that plant communities and deer densities vary temporally. Thus, analysing the temporal aspect of plant community assembly could improve conclusions about the interaction between deer and plant invasion since legacy effects play out over decadal time frames (Royo *et al.* 2010b; Nuttle *et al.* 2011; White 2012). Nonetheless, because sampling was spread across a wide range of sites and years, we expect observed patterns to be robust. We analysed equal numbers of deer-exclusion and deer-access plots from each data set (Table 1) and weighted plots equally.

We analysed plant species presence/absence and two abundance metrics, cover and stem density. Across experiments, plant abundance was quantified in several ways, including stem density (14 sites), per cent cover (8 sites), cover classes (i.e. ranges of per cent cover; 15 sites) and/or density classes (i.e. ranges of population density; 7 sites) (Table 1; for ranges of cover classes used and for treatment of density classes, **see Supporting Information—Fig. S1, Text S1**). We converted cover data to cover classes (for detailed processing methods, **see Supporting Information—Text S1**) and then used midpoints of cover classes (e.g. the midpoint of a 5–25 % cover class is 15 %) in analyses (hereafter referred to as cover).

Some plant species in almost every data set were unidentified and marked as unknown at genus, family or growth habit level (e.g. forb, fern, graminoid, woody seedling). We excluded these from analyses that required knowledge of native/introduced status, but otherwise included them in indicator species analyses and when determining total plot species richness and abundance. We statistically tested effects of deer access/exclusion and deer density on richness and abundance of unknown species **[see Supporting Information—Table S2]**. We standardized species taxonomy and native/introduced status according to the United States Department of Agriculture Plants Database (USDA NRCS 2012). Taxa with status code of ‘Native and Introduced’ (i.e. some infra-taxa are native and some are introduced) were classified as native. We define introduced plants as invasive according to the USDA Forest Service (1998) and the USDA NRCS (2012).

### Statistical analysis

We used mixed effects linear regression to test for effects of deer on relationships between native and introduced species richness and abundance. Introduced vegetation (i.e. species richness, cover or density) was the response variable, native vegetation and deer access/exclusion or deer population density were the fixed effects, and plot pair and site were the random effects. For the native vegetation effect in these models, native cover was used in the introduced cover analysis, native stem density in the introduced stem density analysis, and native species richness in the introduced species richness analysis. Deer density analyses were utilized only for unfenced, deer-access plot data here and below.

#### Deer effects on floristic composition, diversity and community-level abundance.

We used the multi-response permutation procedure (MRPP) (Mielke and Berry 2007) to test for community-level differences in floristic composition between deer-access and deer-exclusion plots using the Sørensen (Bray–Curtis) distance measure, which is not likely to exaggerate the influence of outliers in heterogeneous data, with PC-ORD software (McCune and Grace 2002). We conducted separate MRPPs for presence/absence and each abundance metric, cover and density. We calculated Shannon diversity (*H′*) (Shannon 1948), a combined measure of species richness and relative abundance (Hill 1973; McCune and Grace 2002), for each plot where at least one species was present. We calculated *H′* for each abundance metric to determine floristic diversity using the equation:

$$H2=-\sum_{i=1}^{S}p_{i}1n\;p_{i}$$

where *S* is the total number of species measured according to each abundance metric and *p*_*i*_ is the proportional abundance of species *i* in the plot.

We used linear mixed effects analysis of variance (ANOVA) to test for effects of deer access/exclusion and linear mixed effects regression to test for effects of deer population density on plant richness and absolute abundance of (i) native species, (ii) introduced species and (iii) total species (native plus introduced plus unknown species) and on Shannon diversity. Deer access/exclusion (fencing treatment) or deer population density were fixed effects and plot pair and site were random effects. We evaluated both absolute and proportion of introduced plant abundance (i.e. relative abundance) and plant species richness because they represent different indices of plant invasion; the former represents actual introduced plant abundance/species richness, while the latter represents the portion of plant community abundance/species richness composed of introduced plants. Absolute introduced plant abundance/species richness was evaluated based on the main effect of deer and proportion introduced plant abundance/species richness was evaluated based on the interaction of the deer effect with total vegetation. A significant interaction indicates that the ratio of introduced to total plant abundance/species richness (i.e. proportion introduced) varies with the deer effect. The ratio of introduced to native vegetation provides another index of plant invasion and was evaluated by testing the interaction of the deer effect with native vegetation. A significant interaction would indicate that the ratio of introduced to native vegetation varies with the deer effect. Total cover could exceed 100 % due to overlapping leaves of different species. We excluded sites lacking introduced plants from community-level mixed model analyses with introduced plants in the response variable.

We acknowledge that accurate deer density estimation is particularly difficult in forests (Putman *et al.* 2011). Total population counts can underestimate the actual number of deer by a factor of four or more (Andersen 1961). While distance sampling (Buckland *et al.* 1993, 2001), used to inform many of the estimates included in analyses here **[see Supporting Information—Table S1]**, is a more accurate sampling approach vs. total population counts, considerable error surrounds single estimates and cannot fully account for season-to-season or year-to-year fluctuations or legacy effects of previous deer populations. Additionally, only deer-access plot data were used in these analyses; thus, the paired-plot baseline provided by fenced-plot data is lacking. Due to these limitations, we exercised caution in interpreting results of regression analyses. All sites were included in deer density analyses except West Point, for which deer population density estimates were unavailable.

#### Deer effects on individual introduced and native species’ abundance.

To follow up the MRPP and determine which species might be driving community-level differences, we used indicator species analysis (ISA) to test for species and genera affinities for deer access or deer exclusion (Dufrêne and Legendre 1997). The ISA results show which plant species or genera associate with deer-access or with deer-exclusion plots. We calculated indicator values for each species by multiplying the relative abundance across all plots by the relative frequency across plots within each treatment. We used a Monte Carlo randomization test to determine significance of indicator values, which range from 0 (not detected) to 100 (exclusive association). We conducted separate ISAs for presence/absence, cover and density data. We used PC-ORD software for the ISAs (McCune and Grace 2002). We report species as significantly associated with a treatment when *α* < 0.05.

We used linear mixed effects ANOVA to test the main effect of deer access/exclusion on abundance of individual introduced and native plant species. We conducted these species-level abundance analyses for the most frequently occurring introduced plants (defined here as species present in >5 % of all plots; a total of 13 introduced species) and for the 20 most frequent native plant species (present in >12 % of all plots). Plot pair and site were random effects included in models to control for within- and between-site variability, respectively.

Non-linearities are pervasive in ecology (e.g. Lockwood *et al.* 2013; Turner and Gardner 2015), yet we did not analyse them in the data presented here, opting instead to transform the data and test for linear patterns. For community- and species-level mixed models, we used square root or natural log transformations of response variables when necessary to meet statistical assumptions of normality and homogeneity of residuals. In community-level analyses, we report 95 % confidence intervals for significant fixed effects (*α* < 0.05) and, for mixed effect models with a significant deer treatment effect (*α* < 0.05), we determined least square means using *t*-tests (based on the Satterthwaite approximation for denominator degrees of freedom). To determine significance of random factors, we used log-likelihood ratio tests (chi-square with one degree of freedom, i.e. one effect tested at a time). We used the lme4 (Bates *et al.* 2015), lmerTest (Kuznetsova *et al.* 2013) and vegan (Oksanen *et al.* 2013) packages for mixed model analyses in R version 3.1.2 (R Development Core Team 2014). We report plot-level means and standard errors.

## Results

We recorded 50 introduced and 345 native species in the regional forest understory species pool. Fifty-four species, six of which were introduced, only occurred in deer-access plots. In contrast, 72 species, 16 of which were introduced, only occurred in deer-exclusion plots. Of the introduced species, 32 % occurred only in deer-exclusion plots; 16 % of native species occurred only in deer-exclusion plots. A higher proportion of native species occurred in both deer-access and deer-exclusion plots (70 %) than of introduced species (56 %). Introduced and native species richness and abundance were significantly positively correlated (Table 2; **see Supporting Information—Fig. S2**). We detected no effect of deer on the ratio of introduced to native vegetation (non-significant interactions between deer effect and native species vegetation) (Table 2). At five sites, no introduced species were observed. Total species richness was 23 % higher at sites where introduced species were present vs. where they were absent. For species richness and abundance by deer access/exclusion treatment and site, **see Supporting Information—Tables S4 and S5**, respectively.

**Table 2.** Mixed model effects of white-tailed deer a) access/exclusion and b) population density and native vegetation on introduced plant richness and abundance (per cent cover and stem density)^a^. Results are based on floristic composition data collected from deer-access (unfenced) and deer-exclusion (fenced) plots at 23 sites in east-central and north-eastern USA. The ratio of introduced to native vegetation was evaluated based on the interaction of the deer effect with native vegetation; the lack of significant interactions indicates that the ratio of introduced to native vegetation does not vary with the deer effect. **See Supporting Information—Fig. S2** for the relationships between introduced and native vegetation. For random effect results, **see Supporting Information—Table S3**. *P* values are in bold print if significant at the alpha level *α* < 0.05.

	Introduced species richness	Introduced cover	Introduced stem density
a) Deer access/exclusion			
Intercept (SE)	0.8 (0.2)	1.3 (0.2)	1.2 (0.3)
DA/DE coefficient (SE)	–0.01 (0.08)	–0.2 (0.1)	0.05 (0.1)
*F* statistic (DFn,DFd)	0.028 (1,222)	2.2 (1,193)	0.22 (1,171)
*P* value	0.9	0.1	0.6
Native vegetation coefficient (SE)	0.036 (0.006)	0.012 (0.005)	0.020 (0.006)
*F* statistic (DFn,DFd)	39 (1,388)	11 (1,346)	9.3 (1,257)
*P* value	**<0.001**	**<0.001**	**0.002**
DA/DE * Native vegetation coefficient (SE)	–5 × 10^−4^ (0.005)	–0.003 (0.004)	–0.010 (0.007)
*F* statistic (DFn,DFd)	0.008 (1,216)	0.56 (1,228)	1.9 (1,194)
*P* value	0.9	0.4	0.2
*n*	404	392	290
# Sites	18	17	11
b) Deer density			
Intercept (SE)	1.3 (0.3)	1.1 (0.6)	0.5 (0.7)
DD coefficient (SE)	0.003 (0.005)	–3 × 10^−4^ (0.01)	0.01 (0.01)
*F* statistic (DFn,DFd)	0.54 (1,23)	0.0011 (1,16)	1.4 (1,11)
*P* value	0.5	1	0.3
Native vegetation coefficient (SE)	0.013 (0.008)	0.023 (0.009)	0.03 (0.02)
*F* statistic (DFn,DFd)	2.3 (1,154)	6 (1,127)	2.8 (1,140)
*P* value	0.1	**0.01**	0.09
DD * Native vegetation coefficient (SE)	3 × 10^−4^ (2 × 10^−4^)	1 × 10^−4^ (3 × 10^−4^)	–3 × 10^−4^ (2 × 10^−4^)
*F* statistic (DFn,DFd)	2.4 (1,185)	0.32 (1,177)	1.7 (1,139)
*P* value	0.1	0.6	0.2
*n*	190	184	145
# Sites	17	16	11

^a^Native species richness was used as the native vegetation predictor variable for introduced species richness and native species cover and stem density were used as the native vegetation predictor variables for introduced cover and stem density, respectively. Square-root transformations of species richness and natural log +1 transformations of species cover and stem density were used to meet statistical assumptions. SE = standard error; DA = deer access; DE = deer exclusion; DFn = degrees of freedom, numerator; DFd = degrees of freedom, denominator; *n* = number of observations; DD = deer density.

### Deer effects on floristic composition, diversity and community-level abundance

**Figure 2.**
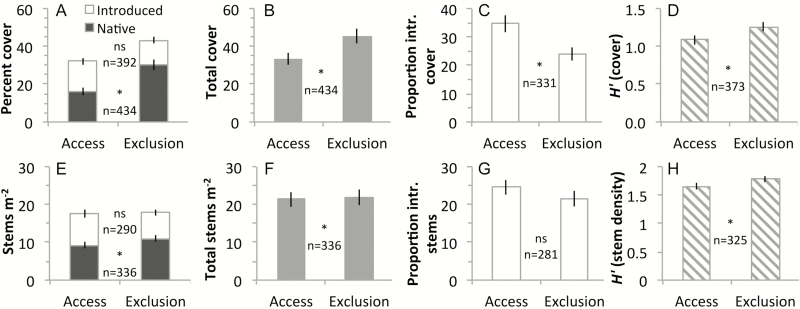
Effects of white-tailed deer access/exclusion on (A, E) introduced and native plant abundance, (B, F) total plant abundance (includes unknown species), (C, G) proportion of introduced (intr.) plants and (D, H) Shannon Diversity (*H′*) in east-central and north-eastern USA. Means (±SE) are presented according to the abundance metric used for data collection, stem density (A–D) and/or cover (E–H) (see Table 1 for additional site information). An asterisk between bars indicates a significant effect of deer; ns = not significant; *n* = sample size (number of plots). The number of plots was constrained in the proportion introduced richness and Shannon diversity analyses due to plots with zero vegetation **[see Supporting Information—Text S1]**.

**Figure 3.**
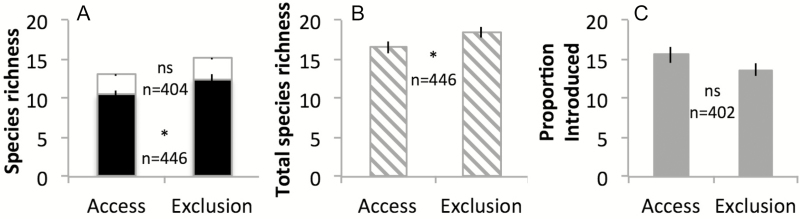
Effects of white-tailed deer access/exclusion on mean (±SE) (A) introduced (white shading) and native (black shading) plant species richness, (B) total plant species richness (includes unknown species) and (C) proportion introduced plant species richness in east-central and north-eastern USA. An asterisk between bars indicates a significant effect of deer; ns = not significant; *n* = sample size (number of plots). The number of plots was constrained in the proportion introduced richness analysis due to plots with zero vegetation **[see Supporting Information—Text S1]**.

Species composition was significantly different between deer-access and deer-exclusion plots based on all three MRPP analyses despite high heterogeneity among plots within each treatment (Table 3). Deer-access plots had lower Shannon diversity (*H′*) than deer-exclusion plots (Table 4a; Fig. 2) and, among deer-access plots, *H′* (cover but not density) was negatively correlated with deer density (Table 4b). Deer exclusion did not affect introduced plant species richness or the proportion of introduced plant species (non-significant interaction between deer access/exclusion and total species richness) (Table 5a; Fig. 3). However, as deer density increased, the proportion of introduced species increased (significant interaction between deer density and total species richness) (Table 5b). Deer-access plots had 16 % lower native plant species richness and 10 % lower total plant species richness than deer-exclusion plots (Table 5a; Fig. 3).

**Table 3.** Results of MRPPs, testing the effect of white-tailed deer on species composition in east-central and north-eastern USA. Separate analyses were conducted for species presence/absence and abundance, per cent cover or stem density. The agreement statistic, *A*, indicates within-group homogeneity compared to random; *A* varies between 0 (heterogeneous plots) and 1 (homogenous plots). The *P* value and the number of plots within each group, deer access or deer exclusion, are shown. The number of plots was constrained in analyses due to plots with zero vegetation **[see Supporting Information—Text S1]**.

	*A*	*P* value	Number of plots	
			Deer access	Deer exclusion
Presence/absence	0.0019	<0.001	221	223
Abundance (cover)	0.0027	<0.001	185	188
Abundance (density)	0.0020	0.001	158	167

**Table 4.** Mixed model effects of white-tailed deer a) access/exclusion and b) population density on introduced, native and total plant density, cover and Shannon diversity (*H*′) based on floristic composition data collected from deer-access and deer-exclusion plots in east-central and north-eastern USA^a^. Proportion introduced plant abundance was evaluated based on the interaction of the deer effect with total vegetation; a significant interaction indicates that the ratio of introduced to total plant abundance (i.e. proportion introduced) varies with the deer effect. The number of plots was constrained in the Shannon diversity analyses due to plots with zero vegetation **[see Supporting Information—Text S1]**. For random effect results, **see Supporting Information—Table S3**. *P* values and LSmeans treatment test results are in bold print if significant at the alpha level *α* < 0.05.

Community index	Introduced cover	Native cover	Total cover	Shannon diversity (cover)	Introduced stem density	Native stem density	Total stem density	Shannon diversity (density)
a) Deer access/exclusion								
Intercept (SE)	0.4 (0.2)	1.9 (0.3)	2.6 (0.3)	1.0 (0.2)	0.8 (0.2)	1.8 (0.2)	2.3 (0.3)	1.6 (0.2)
DA/DE coefficient (SE)	–0.01 (0.1)	0.62 (0.08)	0.3 (0.1)	0.17 (0.05)	0.03 (0.1)	0.30 (0.06)	0.27 (0.06)	0.19 (0.04)
*F* statistic (DFn,DFd)	0.02 (1,201)	58 (1,216)	9.4 (1,216)	12 (1,194)	0.13 (1,161)	23 (1,167)	19 (1,167)	17 (1,161)
*P* value	0.9	**<0.001**	**0.002**	**<0.001**	0.7	**<0.001**	**<0.001**	**<0.001**
LSmeans treatment test	–	**DE > DA**	**DE > DA**	**DE > DA**	–	**DE > DA**	**DE > DA**	**DE > DA**
DA estimate (LCI–UCI)	–	1.9 (1.1–2.6)	2.6 (2.0–3.3)	1.2 (0.8–1.5)	–	1.8 (1.3–2.2)	2.3 (1.7–2.9)	1.6 (1.2–2.0)
DE estimate (LCI–UCI)	–	2.5 (1.8–3.2)	3.0 (2.4–3.6)	1.3 (1.0–1.7)	–	2.1 (1.6–2.5)	2.6 (1.9–3.2)	1.8 (1.4–2.2)
Total vegetation coefficient (SE)	0.032 (0.002)	–	–	–	0.025 (0.002)	–	–	–
*F* statistic (DFn,DFd)	397 (1,343)	–	–	–	95 (1,225)	–	–	–
*P* value	**<0.001**	–	–	–	**<0.001**	–	–	–
DA/DE * Total vegetation coefficient (SE)	–0.011 (0.002)	–	–	–	–0.003 (0.003)	–	–	–
*F* statistic (DFn,DFd)	42 (1,232)	–	–	–	1.1 (1,178)	–	–	–
*P* value	**<0.001**	–	–	–	0.3	–	–	–
*n*	392	434	434	373	290	336	336	325
# Sites	17	22	22	22	11	14	14	14
b) Deer density								
Intercept (SE)	0.2 (0.3)	3.0 (0.4)	3.4 (0.4)	1.7 (0.2)	–0.3 (0.5)	1.8 (0.5)	2.2 (0.6)	1.7 (0.4)
DD coefficient (SE)	0.001 (0.005)	–0.030 (0.009)	–0.020 (0.009)	–0.015 (0.005)	0.018 (0.008)	4 × 10^−5^ (0.008)	0.002 (0.01)	–0.002 (0.006)
*F* statistic (DFn,DFd)	0.081 (1,17)	11 (1,18)	5.3 (1,17)	9.8 (1,18)	4.7 (1,12)	2.9 × 10^−5^ (1,12)	0.026 (1,12)	0.13 (1,12)
*P* value	0.8	**0.004**	**0.03**	**0.006**	0.051	1	0.9	0.7
Total vegetation coefficient (SE)	0.026 (0.003)	–	–	–	0.06 (0.01)	–	–	–
*F* statistic (DFn,DFd)	78 (1,105)	–	–	–	41 (1,126)	–	–	–
*P* value	**<0.001**	–	–	–	**<0.001**	–	–	–
DD * Total vegetation coefficient (SE)	2.6 × 10^−4^ (6 × 10^−5^)	–	–	–	–5 × 10^−4^ (1 × 10^−4^)	–	–	–
*F* statistic (DFn,DFd)	21 (1,152)	–	–	–	18 (1,130)	–	–	–
*P* value	**<0.001**	–	–	–	**<0.001**	–	–	–
*n*	184	205	205	173	145	168	168	158
# Sites	16	21	21	21	11	14	14	14

^a^Natural log +1 transformations of cover and stem density data were used to meet statistical assumptions. SE = standard error; DA = deer access; DE = deer exclusion; DFn = degrees of freedom, numerator; DFd = degrees of freedom, denominator; LCI = lower confidence interval; UCI = upper confidence interval; DD = deer density; *n* = number of observations.

**Table 5.** Mixed model effects of white-tailed deer a) access/exclusion and b) population density on introduced, native and total plant species richness based on floristic composition data collected from deer-access (unfenced) and deer-exclusion (fenced) plots at 23 sites in east-central and north-eastern USA^a^. Proportion introduced plant species richness was evaluated based on the interaction of the deer effect with total species richness; a significant interaction indicates that the ratio of introduced to total plant species richness (i.e. proportion introduced) varies with the deer effect. For random effect results, **see Supporting Information—Table S3**. *P* values and LSmeans treatment test results are in bold print if significant at the alpha level *α* < 0.05.

	Introduced species richness	Native species richness	Total species richness
a) Deer access/exclusion			
Intercept (SE)	0.4 (0.2)	3.2 (0.2)	4.0 (0.3)
DA/DE coefficient (SE)	–0.04 (0.07)	0.39 (0.06)	0.32 (0.06)
*F* statistic (DFn,DFd)	0.31 (1,220)	46 (1,222)	25 (1,222)
*P* value	0.6	**<0.001**	**<0.001**
LSmeans treatment test	–	**DE > DA**	**DE > DA**
DA estimate (LCI–UCI)	–	3.2 (2.7–3.8)	4.0 (3.4–4.5)
DE estimate (LCI–UCI)	–	3.6 (3.1–4.1)	4.3 (3.7–4.8)
Total species richness coefficient (SE)	0.044 (0.004)	–	–
*F* statistic (DFn,DFd)	160 (1,397)	–	–
*P* value	**<0.001**	–	–
DA/DE * Total species richness coefficient (SE)	2 × 10^−4^ (0.003)	–	–
*F* statistic (DFn,DFd)	0.004 (1,219)	–	–
*P* value	0.9	–	–
*n*	404	446	446
# Sites	18	23	23
b) Deer density			
Intercept (SE)	0.2 (0.4)	3.9 (0.4)	4.5 (0.5)
DD coefficient (SE)	0.005 (0.007)	–0.020 (0.009)	–0.01 (0.01)
*F* statistic (DFn,DFd)	0.64 (1,22)	5.1 (1,19)	2.1 (1,19)
*P* value	0.4	**0.04**	0.2
Total species richness coefficient (SE)	0.031 (0.008)	–	–
*F* statistic (DFn,DFd)	15 (1,183)	–	–
*P* value	**<0.001**	–	–
DD * Total species richness coefficient (SE)	3 × 10^−4^ (1 × 10^−4^)	–	–
*F* statistic (DFn,DFd)	3.9 (1,185)	–	–
*P* value	**0.049**	–	–
*n*	190	211	211
# Sites	17	22	22

^a^Square-root transformations of species richness were used to meet the assumption of homogeneity of residuals. SE = standard error; DA = deer access; DE = deer exclusion; DFn = degrees of freedom, numerator; DFd = degrees of freedom, denominator; LCI = lower confidence interval; UCI = upper confidence interval; *n* = number of observations; DD = deer density.

While deer-access plots tended to have higher absolute introduced plant abundance than deer-exclusion plots, these trends were not statistically significant (Fig. 2; Table 4). However, in deer-access plots, native stem density was 16 % lower and native cover was 46 % lower than in deer-exclusion plots (Fig. 2; Table 4). Total stem density was 2 % lower and total cover was 27 % lower in deer-access plots than in deer-exclusion plots. The proportion of introduced plant cover was 44 % higher in deer-access vs. deer-exclusion plots (Fig. 2; Table 4a). The proportion of introduced plant stems was numerically, but not statistically, higher in deer-access plots (Fig. 2; Table 4a). The proportion of introduced plant abundance (cover and stem density) was positively correlated with deer density, while native and total plant cover were negatively correlated with deer density (Table 4b). In summary, deer had no effect on absolute introduced abundance but they increased the proportion composed of introduced species. The difference between the absolute and proportional metrics is native vegetation, which is reduced by deer. Thus, deer indirectly increase the proportion of introduced vegetation via their negative influence on native vegetation. Notably, we detected these effects after accounting for within- and between-site variability, which were significant random factors **[see Supporting Information—Table S3]**. More unknown species occurred in deer-access plots than in deer-exclusion plots but unknown species abundance was unaffected **[see Supporting Information—Table S2]**.

### Deer effects on individual introduced and native species’ abundance

Deer access/exclusion differentially affected introduced and native plant species. Indicator species analysis results showed that three introduced species and four native species were indicators of deer-access plots, while three introduced and 15 native species were indicators of deer-exclusion plots (Table 6). Two introduced plants, the grass *Microstegium vimineum* and the herb *Alliaria petiolata*, occurred in a large number of plots and sites and were the best indicator species (by indicator value) of deer-access plots (Table 6). Indicator species of deer-exclusion plots included the introduced vine *Lonicera japonica* and shrub *Rosa multiflora* (Table 6). In general, introduced indicator species were found to be more abundant in their respective deer-access or deer-exclusion plots using mixed model analyses (Table 7). In deer-access plots, absolute abundance of three introduced species, *M. vimineum*, *A. petiolata* and the tree *Ailanthus altissima*, was higher than in deer-exclusion plots. In contrast, three other introduced species, *L. japonica*, *R. multiflora* and *Duchesnea indica*, occurred in lower abundance in deer-access vs. deer-exclusion plots (Table 7).

**Table 6.** Indicator species analysis results showing plant species and genera associated with deer access or with deer exclusion in east-central and north-eastern USA. Introduced species are in bold type. Indicator values range from 0 (no indication of association with treatment) to 100 (perfect indication) and were determined according to species’ presence/absence (p/a) and the metric used to record abundance, density and/or cover. The number of plots and sites where each species was observed is included to indicate frequency and distribution across the 23 sites analysed. Results are arranged by deer access/exclusion, then by indicator value and then by *P* value; each species’ results are listed together.

	Species	Habit^a^	Abundance measurement	Indicator value	*P* value	# Plots	# Sites
Deer access	****Microstegium vimineum****	Graminoid	Cover	35	<0.001	146	13
			p/a	23	0.02	148	14
	****Alliaria petiolata****	Forb/herb	Density	29	0.02	133	9
	*Polygonum*		Density	13	0.05	47	7
	*Pilea pumila*	Forb/herb	Density	11	0.01	33	5
			p/a	8	0.04	40	9
	*Oxalis*		Density	8	0.04	26	6
	*Oxalis stricta*	Forb/herb	Density	8	0.02	21	4
	****Perilla frutescens****	Forb/herb	p/a	6	0.04	26	7
	*Acalypha rhomboidea*	Forb/herb	Density	5	0.009	9	2
			p/a	3	0.02	9	2
	*Cinna arundinacea*	Graminoid	Cover	3	0.03	7	2
	*Prenanthes*		Cover	3	0.04	7	3
	*Solanum*		Density	3	0.05	4	4
Deer exclusion	****Lonicera japonica****	Vine	Density	25	0.05	118	9
			Cover	16	0.01	67	6
	*Parthenocissus quinquefolia*	Vine	Cover	25	0.01	111	11
	*Prunus serotina*	Tree, shrub	p/a	24	0.009	159	19
	*Toxicodendron radicans*	Shrub, forb/herb, subshrub, vine	Density	20	0.04	86	9
	****Rosa multiflora****	Vine, subshrub	p/a	14	0.03	81	11
			Cover	8	0.03	28	6
	*Maianthemum racemosum*	Forb/herb	Cover	13	<0.001	42	12
			p/a	12	0.007	61	16
			Density	8	0.02	23	5
	*Ulmus rubra*	Tree	Density	13	0.008	45	8
			p/a	12	0.005	56	9
	*Rubus*		p/a	13	0.03	77	13
	*Viburnum acerifolium*	Shrub, subshrub	Cover	11	0.001	28	8
			p/a	10	0.001	37	10
	*Carya cordiformis*	Tree	Density	11	0.004	31	8
			p/a	10	0.01	47	13
	*Quercus rubra*	Tree	Density	11	0.01	33	7
	*Polygonatum biflorum*	Forb/herb	Cover	10	0.03	30	10
			p/a	8	0.02	37	13
	*Carya alba*	Tree	p/a	6	0.03	28	10
			Density	6	0.03	17	7
			Cover	5	0.05	16	4
	*Actaea racemosa*	Forb/herb	Cover	5	0.03	12	5
	*Rhododendron periclymenoides*	Shrub	p/a	4	0.02	10	4
			Cover	3	0.03	6	2
	*Euthamia graminifolia*	Forb/herb	Cover	3	0.03	6	1
			p/a	3	0.03	6	1
	*Circaea alpina*	Forb/herb	Cover	3	0.03	6	1
			p/a	3	0.03	6	1
	****Lonicera maackii****	Shrub	p/a	3	0.01	7	3
	*Rubus pensilvanicus*	Subshrub	p/a	3	0.03	6	3

^a^The native status based on genus alone is unknown.

**Table 7.**
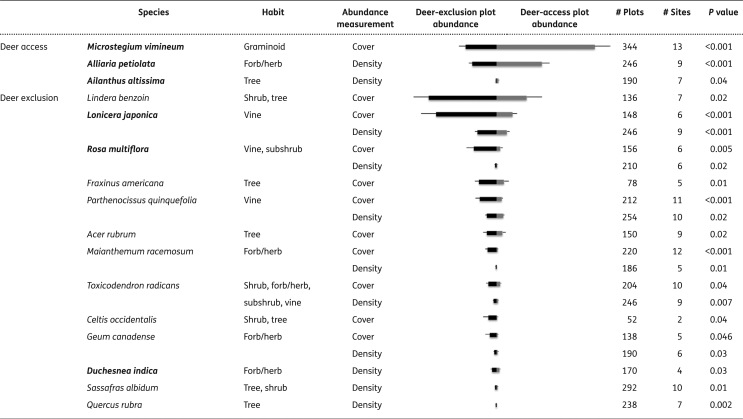
Effects of deer on the abundance of the most frequent introduced (in bold type) and native plant species in east-central and north-eastern USA based on mixed models using floristic composition data collected from deer-access and deer-exclusion plots. Only significant effects are shown of the 13 introduced and 20 native species analysed. Abundance (+SE) in deer-access and deer-exclusion plots is presented; units for density are plants m^−2^ and for cover are per cent cover. Results are arranged by deer access/exclusion and then by abundance; each species’ results are listed together.

The native herbs *Pilea pumila* and *Oxalis stricta* were indicators of deer access, but others, including *Maianthemum racemosum* and *Polygonatum biflorum* were indicators of deer exclusion. Native trees *P. serotina*, *Ulmus rubra*, two *Carya* spp. and *Quercus rubra* and native shrubs *Viburnum acerifolium* and *Rhododendron periclymenoides* were indicator species of deer exclusion (Table 6). The cover of native trees *Fraxinus americana*, *Acer rubrum* and *Celtis occidentalis* and the native shrub *Lindera benzoin* was reduced in deer-access plots relative to deer-exclusion plots (Table 7). The native vines *Parthenocissus quinquefolia* and *Toxicodendron radicans* were indicators of deer exclusion and occurred in greater abundance in deer-exclusion vs. deer-access plots (Tables 6 and 7). Unknown species in the genera *Polygonum*, *Oxalis*, native *Prenanthes* and *Solanum* were associated with deer access, while unknown *Rubus* spp. indicated deer exclusion. For frequencies of each taxon recorded in deer-access plots, in deer-exclusion plots and overall, **see Supporting Information—Table S6**.

## Discussion

White-tailed deer alter plant species composition and reduce community-wide plant diversity, upholding our first prediction. Deer facilitate some but not all introduced plant species and strongly negatively affect native plant species, offering partial support to our second prediction that deer would benefit introduced plants and disadvantage native plants. This work substantially clarifies previous conflicting reports of deer effects on introduced plants. By exploring deer-plant patterns across the region, our results provide evidence that attribute the seemingly contradictory findings in individual site-level studies to species-level differences, illustrating the presumed consequences of differential palatability to deer (Senft *et al.* 1987; Keane and Crawley 2002; Averill *et al.* 2016). Our results are consistent with previous research showing that deer can increase (Knight *et al.* 2009; Eschtruth and Battles 2009b; Beasley and McCarthy 2011; Kalisz *et al.* 2014; Dávalos *et al.* 2015b), decrease (Rossell *et al.* 2007; Shelton *et al.* 2014), have no effect (Bowers 1993; Levine *et al.* 2012; DiTommaso *et al.* 2014) or mixed effects (Cadenasso *et al.* 2002; Webster *et al.* 2005; Knapp *et al.* 2008; Shen *et al.* 2016) on introduced plants. Where deer facilitate an increase in introduced plant abundance, plant invasion via enemy release (Elton 1958; Keane and Crawley 2002; Colautti *et al.* 2004) might be responsible. In contrast, where deer decrease introduced plants, biotic resistance to plant invasion is a possible outcome (Levine *et al.* 2004; Parker and Hay 2005; Parker *et al.* 2006). Despite within- and between-site heterogeneity, the fact that deer had negative impacts on native plants and indirect, facilitative effects on the proportion of introduced plant abundance elucidates the overarching effects of deer on vegetation at a regional scale (Russell *et al.* 2001).

That introduced and native species richness and abundance patterns are positively correlated (Table 2; **see Supporting Information—Fig. S2**) is consistent with research showing that introduced plant species invade ‘hot spots’ of diversity at large spatial scales (Stohlgren *et al.* 1999; Stark *et al.* 2006). Site characteristics, such as spatial heterogeneity in abiotic conditions (Davies *et al.* 2005), including land-use history and soil nutrients (Fraterrigo *et al.* 2006) across sites likely are responsible for the positive relationship between native and introduced plant richness and abundance.

### Deer effects on floristic composition, diversity and community-level abundance

Deer do not directly impact introduced plant species richness (Table 5; Fig. 3) or abundance (Table 4; Fig. 2), which is evidence against our second prediction. These results are surprising, as many sites and large areas within the region and across the world currently are dominated by introduced species and also have high deer or other large herbivore populations (Waller and Alverson 1997; Rooney *et al.* 2004; Vavra *et al.* 2007). Such observations prompted our second prediction that greater richness and abundance of introduced plants would accompany deer access vs. deer exclusion. The fact that deer increase the proportion of cover of introduced plants appears to arise from the substantial decrease in the native flora imposed by deer. These results imply that the positive deer effect on the relative cover of introduced plants is caused indirectly by greater susceptibility of native vs. introduced plants to deer (however, see species-level results below). Deer have a markedly stronger negative effect on native species than on introduced species both in forest understories, as found in this work, and in an old field (DiTommaso *et al.* 2014). This result stands in contrast to reports that native and introduced species behave similarly in dynamic systems (Meiners 2007; Stromberg *et al.* 2009), albeit because of a native herbivore. The perspective that species be judged based on function and not on where they originated is gaining ground (Davis *et al.* 2011), yet our results show an important difference between native and introduced plants, namely their general susceptibility or response to herbivory, suggesting that native status has a deserved role in future research and in management decision-making. Our analyses suggest that declines in plant community diversity (McKinney and Lockwood 1999; Rooney *et al.* 2004) result more from deer herbivory than from the presence of introduced plants, a result also detected in other work (Morrison, this issue). Deer are a key driver of community change (Waller and Alverson 1997), while invasive plants are likely passengers opportunistically taking advantage of ecosystem alterations (MacDougall and Turkington 2005; Didham *et al.* 2007).

Our finding that deer increase the proportion of cover of introduced plants (Fig. 2) opposes the broadly observed biotic resistance pattern in which native herbivores reduce the relative abundance of introduced vegetation (Parker *et al.* 2006) as a result of differential palatability among introduced and native species (Parker and Hay 2005). This global meta-analysis found that native herbivores (e.g. insects, rodents and cervids) suppress introduced plants more than native plants. While informative for plant–herbivore interactions generally, such an extensive analysis is less likely to be predictive for a particular herbivore. Nonetheless, deer herbivory is a constant and important filter of regional species pools (Rooney *et al.* 2004) and could have a role in biotic resistance for certain introduced species (Maron and Vilà 2001), even preventing them from appearing in floristic census records.

### Deer effects on individual introduced and native species’ abundance

Overall, we found a few graminoid and herbaceous species are favoured in the presence of deer, while trees, shrubs, vines and many herbaceous species lose out (Tables 6 and 7). These findings are consistent with assessments of winning and losing species in Northern Wisconsin (Rooney *et al.* 2004; Wiegmann and Waller 2006) and globally (McKinney and Lockwood 1999). Our finding that many woody and herbaceous plant species are negatively impacted by deer contrasts with results from a meta-analysis showing that woody, but not herbaceous species are negatively impacted by deer (Habeck and Schultz 2015), a discrepancy possibly owing to publication bias detected in the meta-analysis. Our work clearly shows that deer facilitate several notorious invasive plants in east-central and north-eastern USA, including *A. altissima* (tree-of-heaven), *A. petiolata* (garlic mustard) and *M. vimineum* (Japanese stilt-grass) (Tables 6 and 7). Positive effects of deer on *A. petiolata* and *M. vimineum* have been found in site-level experiments (Eschtruth and Battles 2009a, b; Knight *et al.* 2009; Kalisz *et al.* 2014; Dávalos *et al.* 2015a, b) and deer have been implicated in the establishment of *A. altissima* (Knapp and Canham 2000). The facilitative effect of deer on these species is likely due to their unpalatability relative to other plants. In deer preference trials, *A. petiolata* and *M. vimineum* were the least palatable of 15 introduced and native species (Averill *et al.* 2016). *Ailanthus altissima* is apparently also unpalatable (Forgione 1993), yet anecdotal evidence of browsing has been observed (K. L. Caraher, Hood College, pers. obs.) and thus the species’ rapid growth rate (Knapp and Canham 2000) could outweigh herbivory. These results show how unpalatable plants can gain an apparent competitive advantage relative to palatable plants (Holt 1977), i.e. native plants, and become more strongly represented in the flora or even invasive (Senft *et al.* 1987; Keane and Crawley 2002; Royo and Carson 2006; Arcese *et al.* 2014).

While deer facilitated an increase in the abundance of several unpalatable invaders in unfenced plots, deer exclusion in fenced plots resulted in higher abundance of several other invaders, including *L. japonica* (Japanese honeysuckle) and *R. multiflora* (multiflora rose) (Table 7). *Lonicera japonica*, *L. maackii*, and *R. multiflora* were indicator species of deer-exclusion plots (Table 6), reinforcing previous findings (Shelton *et al.* 2014) and suggesting these fleshy-fruited species perform better where protected against deer browsing. Even if species perform well enough where deer occur to be considered invasive, they might perform better where deer are excluded. These findings might be an outcome of one or several processes, three of which are outlined here. (i) These species are palatable (Sheldon and Causey 1974; Ashton and Lerdau 2008; Averill *et al.* 2016) and, in heavily browsed plant communities, the most palatable species are the most susceptible to being consumed and reduced in abundance (Royo and Carson 2006). Indeed, a decrease of *L. japonica* has been observed anecdotally in south-eastern Indiana as deer populations increased from the 1970s through 1990s (D. K. Apsley, The Ohio State University, pers. obs.). Tangentially, palatable invasive shrubs, such as *L. maackii*, which offers a leafy source of protein in early spring when native species are still leafless, might serve to boost deer populations (Martinod and Gorchov, this issue). (ii) Increased propagule pressure via bird-dispersal could account for the higher abundance of fleshy-fruited species observed in fenced plots. Birds are attracted to the additional habitat (e.g. food, shelter and perch points) and fences occurring where deer are excluded (McShea and Rappole 2000; Chollet and Martin 2013) and they are liable to disperse plant seeds via their droppings. Mutualistic interactions are of known importance in plant invasion (Richardson *et al.* 2000; (Gleditsch and Carlo 2011). (iii) Vines, such as *L. japonica* and the native *P. quinquefolia*, could be more abundant in fenced plots because they can climb on the more abundant vegetation occurring in deer-exclusion plots and on the fences themselves. The possibility of climbing was controlled experimentally at the two Valley Forge sites through the use of a metal stake placed in the centre of control plots (Abrams and Johnson 2012), yet the few occurrences of *L. japonica* and *R. multiflora* at the Valley Forge–Mt Joy (VJ) site were in deer-exclusion plots **[see Supporting Information—Table S6]**, implicating deer exclusion as causal in increasing these vines’ abundance.

Deer have strong negative impacts on native species of many life forms. Overstory species, such as *A. rubrum*, *Carya* spp., *F. americana* and *Quercus* spp., appear to benefit from deer exclusion (Tables 6 and 7). Many other researchers (e.g. Abrams and Johnson 2012; Bressette *et al.* 2012; Nuttle *et al.* 2013; Abrams 2013; Owings *et al.*, this issue) also report negative impacts of abundant deer on native tree species, implying that forest regeneration could be at risk. Shrubs, including *L. benzoin*, *R. periclymenoides* and *V. acerifolium*, also appear negatively influenced by deer (Tables 6 and 7), which jeopardizes organisms in other trophic levels that depend on forest understory shrub layers, e.g. birds (deCalesta 1994; McShea and Rappole 2000; Fuller 2001; Chollet and Martin 2013).

### Site influences

Five sites were uninvaded by introduced plants. However, the native fern *Dennstaedtia punctilobula*, which is considered a native invasive plant (de la Cretaz and Kelty 1999), is dominant at two of the sites in north-eastern Pennsylvania (Long Run and Marienville). At the other three sites (Fernow, Monongahela and Zaleski), deer density estimates were considerably lower (~6 deer km^−2^) than the average across sites (mean = 35 deer km^−2^; median = 26 deer km^−2^) (Table 1). At Fernow and Monongahela, deer were shown to increase herbaceous richness and abundance by reducing fast-growing early successional species (Royo *et al.* 2010a). Thus, sites without introduced invaders might instead have native invaders or low deer densities, which might be associated with increased biotic resistance to introduced plant invasion.

In addition to deer density, overstory species composition and duration of deer exclusion varied among sites (Table 1) and likely contributed to varying deer effect patterns at the site level **[see Supporting Information—Tables S4 and S5]**. While sites were not selected randomly, most were not established to study invasive plants and spanned a wide deer abundance gradient (Table 1). Furthermore, many concomitant and often interactive factors not limited to deer and invasive plants (e.g. forest successional age and proximity to centres of human activity, propagule pressure, resource availability, invasive earthworms, etc.) affect forest understory diversity (Abrams 1998; Pysřek *et al.* 2002) Baiser *et al.* 2008; Eschtruth and Battles 2009b; Royo *et al.* 2010a; Martin and Baltzinger 2011; Fisichelli *et al.* 2013; Dávalos *et al.* 2014; Dobson and Blossey 2015; Forsyth *et al.* 2015; Nuzzo *et al.* 2015), yet were not included in analyses here. Including such factors in future work would improve understanding of community assembly and invasion processes. The influence of some site characteristics, including surrounding landscape structure and composition, on the relationship between deer and plant invasion is explored elsewhere (Averill 2014).

In floristic censuses, plant abundance is sometimes sampled using different metrics for different plant habits (Table 1), which presents issues for pooled or meta-analysis, such as requiring analysis and interpretation of multiple abundance metrics. The results reported here also show that using different abundance metrics can yield different results. For example, deer access increased the proportion of introduced plant cover, but not stem density (Table 4; Fig. 2), perhaps because herbivory influences cover more than stem density. Furthermore, determining total stem density or total cover, and therefore total vegetation abundance, depends on species being sampled in the same way. Total plant abundance is a useful metric for relating primary productivity to ecosystem functioning (Chapin *et al.* 2002), but cannot be calculated in data sets that use different abundance metrics for different plant habits, as was the case here.

## Conclusions

This analysis deepens ecological understanding of some key factors in the invasion process. In this work, an abundant, native, large herbivore is shown to alter plant community composition, lower diversity, reduce native plant richness and abundance, and increase the relative cover of introduced plants. Unpalatable invasive plants seem to benefit under heavy herbivore pressure. While introduced plant invasion has been causally implicated in native plant decline (Wiegmann and Waller 2006), ruminant herbivory appears to be a key factor affecting both processes. Dominant native herbivores such as deer are important agents of ecosystem change as their presence (i) reduces native biodiversity and (ii) increases the relative abundance of introduced plants, two of the major drivers affecting modern plant communities and ecosystems (Hooper *et al.* 2012).

## Supporting Information

The following additional information is available in the online version of this article—

**Text S1**. Additional vegetation data processing methods.

**Table S1**. Sources and methods for deer density estimates.

**Table S2**. Statistical results for effects of deer on unknown species.

**Table S3**. Statistical results showing the influence of the random effects plot and site.

**Table S4**. Species richness by plant native status, deer access/deer exclusion and site.

**Table S5**. Vegetation abundance by metric used (per cent cover or stem density), plant native status, deer access/deer exclusion and site.

**Table S6**. Taxa frequency by deer access/deer exclusion and plant native status, with sites of occurrence.

**Figure S1**. Vegetation cover class categories used to estimate plant abundance across 15 sites.

**Figure S2**. Relationships between introduced and native species richness and abundance.

## Sources of Funding

This work was funded by: the United States Department of Agriculture National Needs Program (K.M.A. and D.A.M.), Penn State College of Agricultural Sciences (K.M.A.), National Science Foundation (NSF) awards DEB 1457531 and DEB 0958676 (S.K.), the NSF award DBI 0851303 and DBI 1156799 (J.D.P.), the Cooperative Agreement H399206006 from the National Park Service (J.D.P.), the U.S. Forest Service award RWU NE-4557 (through agreement JV-11242328-121 with Hood College) (D.H.B. and K.L.C.), the U.S. Department of Energy (DOE) Fermilab National Environmental Research Park (operated by Fermi Research Alliance, LLC under Contract No. DE-AC02-07CH11359 with the US DOE) (V.A.N.) and the Strategic Environmental Research and Development Program (SERDP) of the U.S. Department of Defense (Grant RC-1542) (B.B.).

## Contributions by the Authors

K.M.A., D.A.M., E.A.H.S., S.K., W.J.M., N.A.B. and J.D.P. conceived of the project. A.A.R. established Long Run and Marienville deer exclusion experimental sites, which were re-sampled by K.M.A. and D.A.M. K.M.A., D.A.M., S.K., W.J.M., N.A.B., J.D.P., M.D.A., D.K.A., B.B., D.H.B., K.L.C., S.E.J., R.M. and V.A.N. established experiments and/or collected data. S.E.J. created Fig. 1. K.M.A. analysed the data and wrote the manuscript. All authors contributed to revisions.

## Conflicts of Interest

None declared.

## Supplementary Material

Supporting Information
