# Supplementary material for: A regional assessment of white-tailed deer effects on plant invasion
Source: AoB Plants. 2017 Dec 7;10(1):plx047. doi: 10.1093/aobpla/plx047 (PMC5761582; doi:10.1093/aobpla/plx047)
Supplement: Supporting Information [file plx047_suppl_supporting_information.docx]

**SUPPORTING INFORMATION Text S1:** Additional vegetation data processing methods.

Fenced and unfenced plots were paired based on initial investigator-designated pairings or numerically according to plot identification numbers. For datasets provided at the subplot or sample quadrat level, plot-level means were calculated.

Cover data were converted to cover classes using the Fermi/West Point system of cover classes (**[see SUPPORTING INFORMATION Figure S1]**). Cover class data were converted to cover class midpoint data for analyses. Sapling count, i.e., population density, data were combined with seedling count data. At six sites, a density class of “>10 stems” was sometimes used, in which case the most conservative value of “11” was substituted for analyses. Density class data were recorded at one site (SH), so the following conservative conversions to density were used: “2-3 stems” = 2.5 stems; “4-5 stems” = 4.5 stems; and “>5 stems” = 6 stems. Density analyses were conducted and reported on a m^2^-basis.

At three sites (FN, MA, and MG), cover and count were recorded, thus both measures of flora at those sites were included in respective analyses. It follows that the separate plant abundance analyses should be interpreted individually to assess support for the hypotheses. Notably, when the sites, FN, MA, and MG, were excluded from community-level analyses, statistical results were the same as when they were included. At ten sites (e.g., AN, CA, and VJ), different plant habits were recorded with separate abundance measures, for example cover class for the more numerous herbs and grasses and density for less numerous woody species; thus floristic datasets were split accordingly and analyzed only with other data of the same measure. At the remaining sites (Table 1), researchers reported only count or cover data.

The number of plots or sites included in some analyses varied depending on the data recorded in each plot, sometimes resulting in unpaired deer-access and deer-exclusion plots, i.e., uneven sample sizes. In two deer-access plots (Valley Forge–Mt. Joy), zero vegetation was recorded, thus these plots were excluded from MRPP and Shannon diversity analyses and analyses of proportion introduced species richness and abundance. We also excluded plots in which zero vegetation was recorded using a particular abundance metric from MRPP and Shannon diversity analyses and analyses of proportion introduced of that abundance metric. If an abundance metric was not measured at a site, the site was excluded from analysis of that metric.


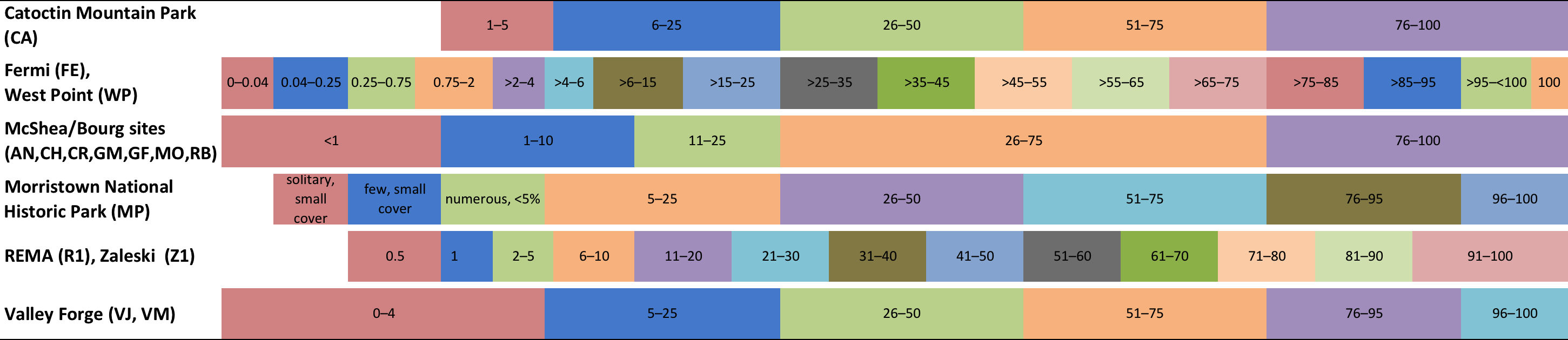


**Supporting information Figure S1:** Vegetation cover class categories used within 15 floristic composition datasets to measure plant abundance in deer-access (unfenced) and deer-exclusion (fenced) plots in east central and northeastern United States.


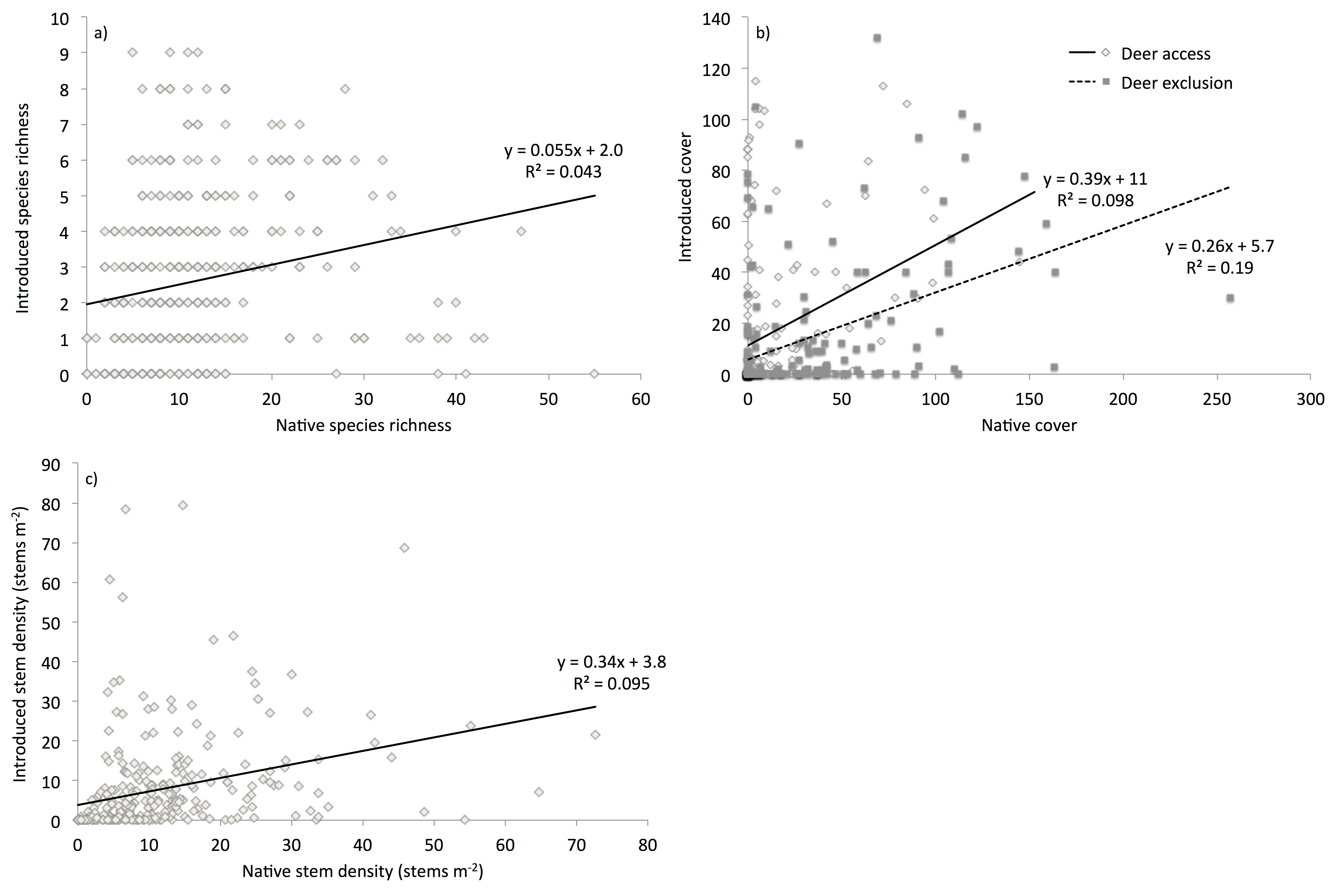


**Supporting Information Figure S2:** Relationships between plot-level native and introduced plant a) species richness and abundance in b) percent cover and c) stem density. Results are based on floristic composition data collected from deer-access (unfenced) and deer-exclusion (fenced) plots in east central and northeastern United States. Linear regression analysis results are shown for significant relationships; however, main statistical analyses included the random effects, plot pair and site (Table 2).

**Supporting information Table S1**: Sources and methods for deer density estimates at sites used in pooled analyses testing the effect of deer on introduced and native plants at 23 sites in north central and northeastern United States.


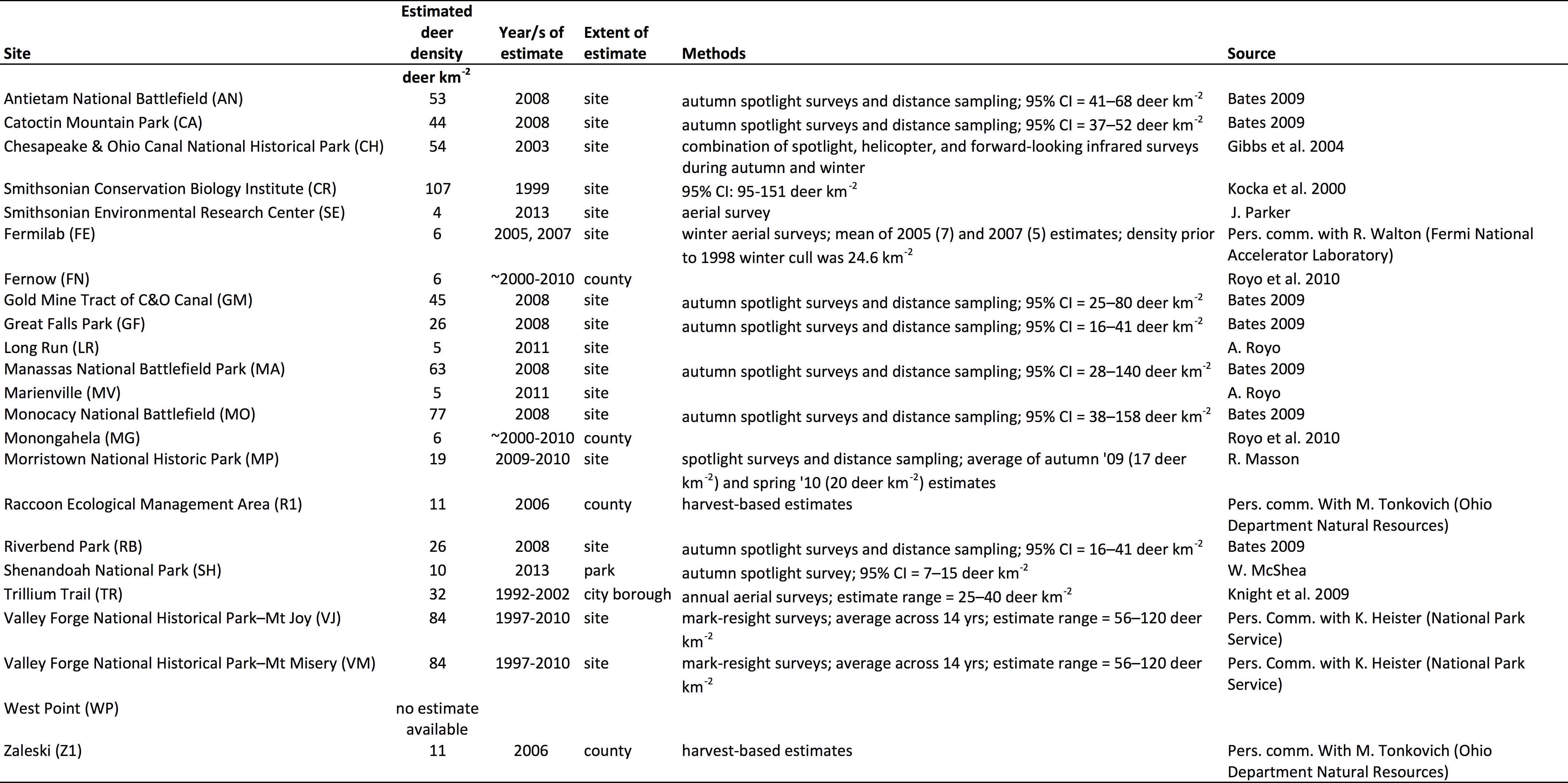


**Supporting information Table S2:** Mixed model effects of white-tailed deer a) access/exclusion and b) population density on unknown vegetation based on floristic composition data collected from deer-access and deer-exclusion plots in east central and northeastern United States^a^.


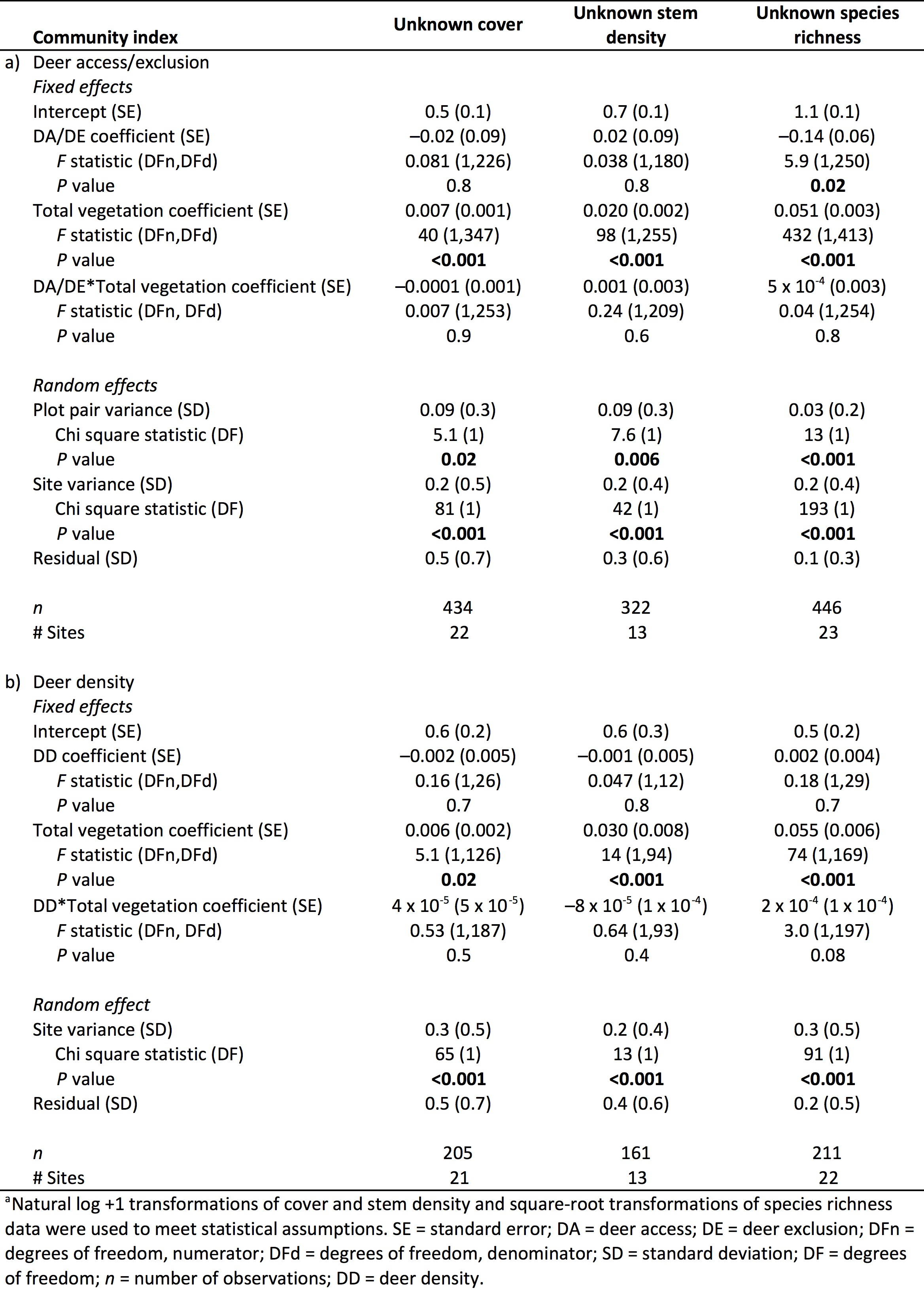
**Supporting information Table S3:** Random effects results from mixed models testing influence of white-tailed deer a) access/exclusion and b) population density on the relationships between introduced and native vegetation, on species abundance and diversity, and on species richness^a^.


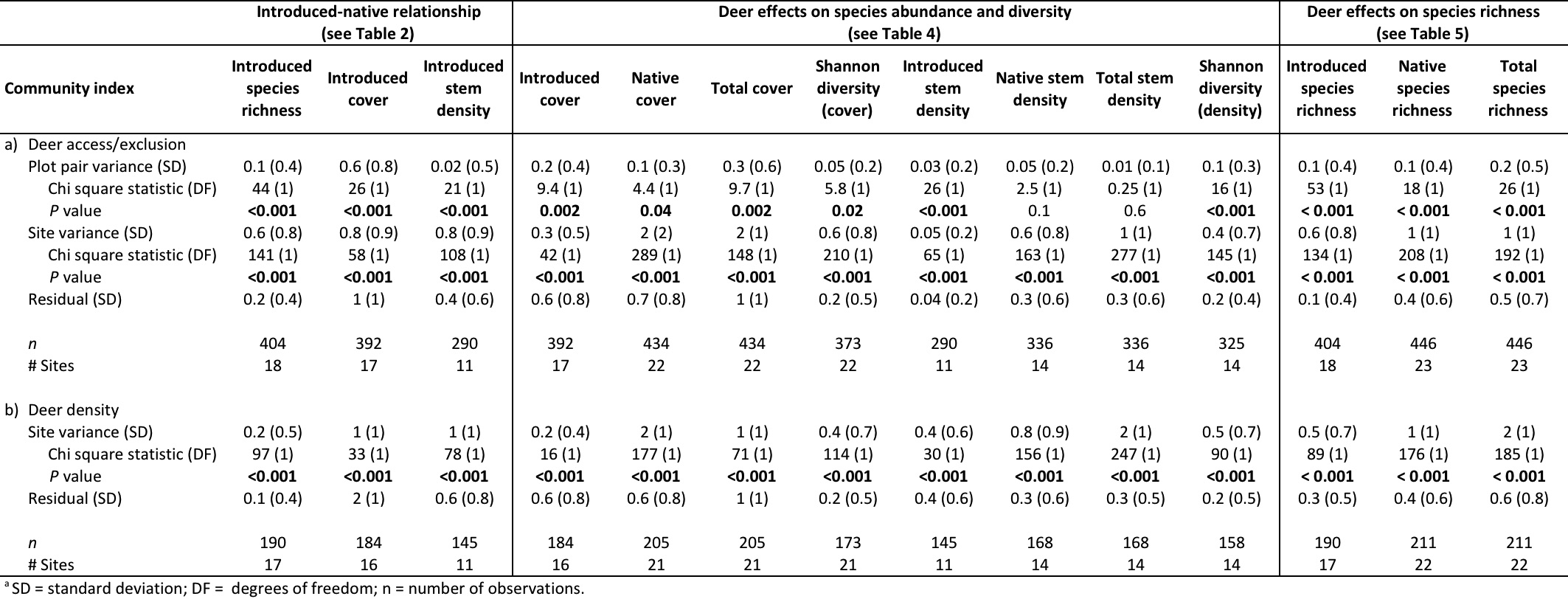


**Supporting information Table S4:** Mean (±SE) species richness according to introduced/native status, deer-access (Deer acc.)/deer-exclusion (Deer excl.), and site. Data were collected from deer-access (unfenced) and deer-exclusion (fenced) plots in east central and northeastern United States. Means across sites are also included.


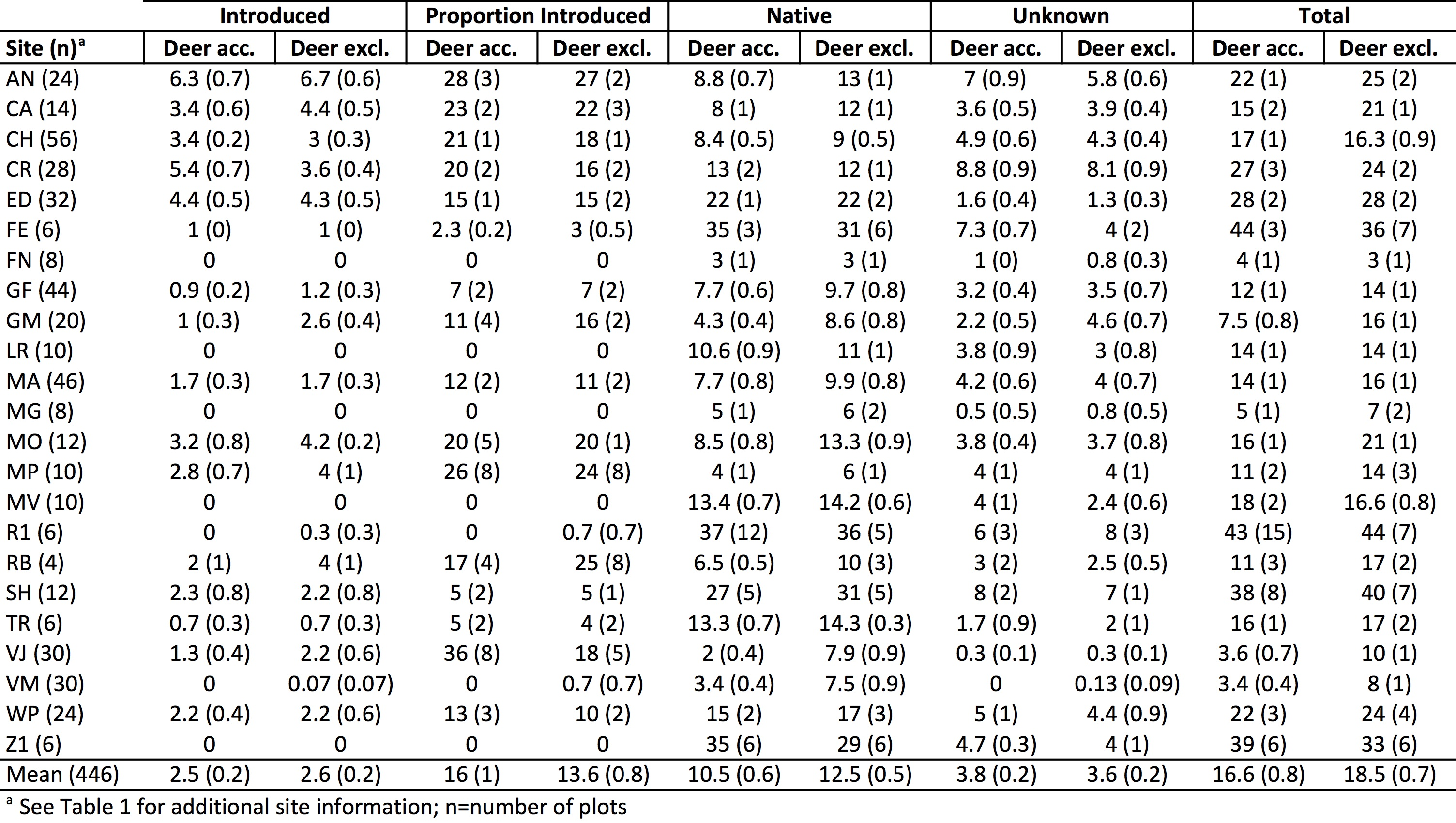


**Supporting information Table S5:** Mean (±SE) vegetation abundance according to metric used (percent cover or stem density), introduced/native status, deer-access (Deer acc.)/deer-exclusion (Deer excl.), and site. Data were collected from deer-access (unfenced) and deer-exclusion (fenced) plots in east central and northeastern United States. Means across sites are also included. Empty cells indicate that particular abundance metric was not measured at the site.


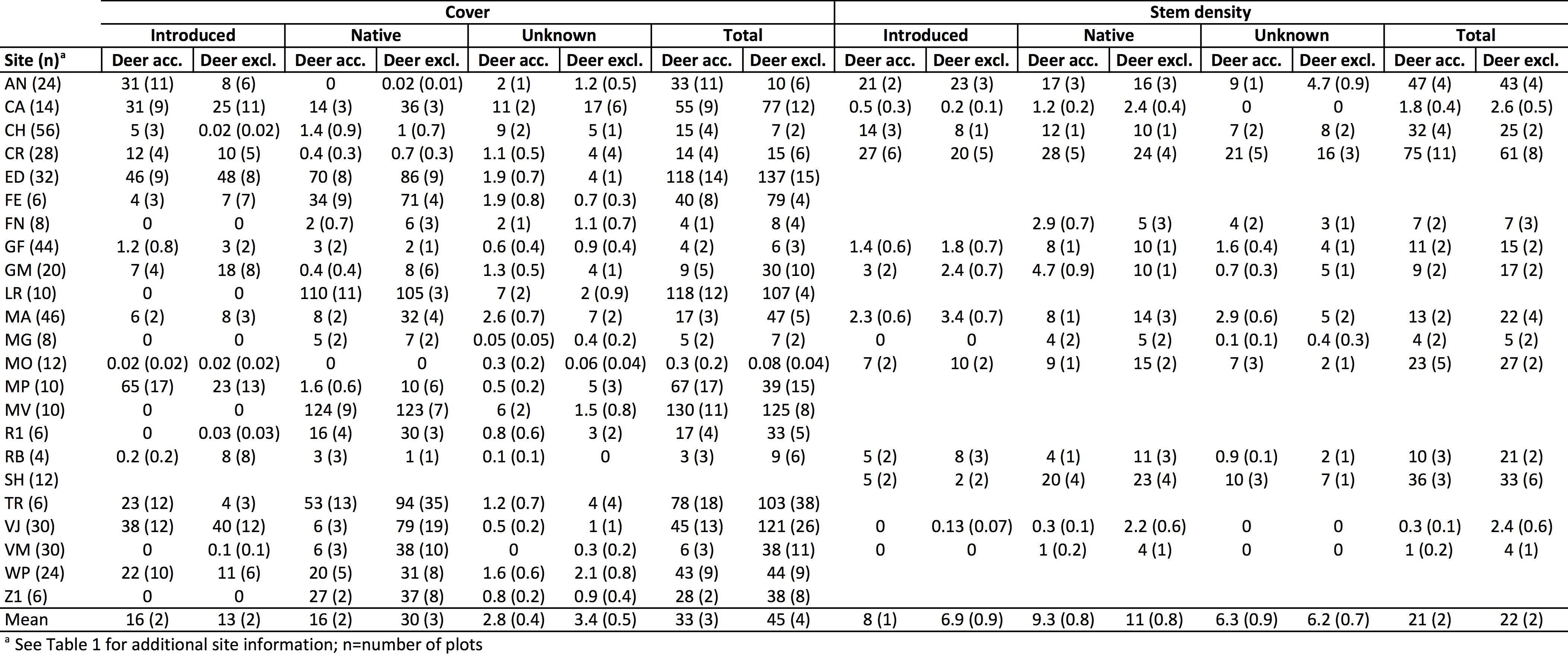


**Supporting information Table S6:** Frequency of each species or taxon recorded in deer-access and deer-exclusion plots and overall across 23 sites in east central and northeastern United States, arranged by introduced/native/unknown status and alphabetically, including the sites where they occurred. Occurrence at a site only in deer-access (DA) or in deer-exclusion (DE) plots is specified.

| **Introduced/ native/ unknown** | **Species** | **Frequency of occurrence** | | | **Sites^a^ where recorded** |
| --- | --- | --- | --- | --- | --- |
|  |  | **Deer-access** | **Deer-exclusion** | **Frequency across all plots** |  |
| Introduced | *Acer platanoides* L. | 0 | 0.013 | 0.007 | VJ(DE) |
|  | *Agrostis gigantea* Roth | 0.004 | 0 | 0.002 | SE(DA) |
|  | *Ailanthus altissima* (Mill.) Swingle | 0.087 | 0.074 | 0.081 | AN, CA, CH(DE), CR, GF(DA), MO, MP(DE), SH |
|  | *Alliaria petiolata* (M. Bieb.) Cavara & Grande | 0.371 | 0.367 | 0.369 | AN, CA, CH, CR, FE, GF, GM, MA, MO, MP, RB, SH, TR, VJ, WP |
|  | *Allium vineale* L. | 0.004 | 0.004 | 0.004 | AN |
|  | *Anthoxanthum odoratum* L. | 0 | 0.004 | 0.002 | WP(DE) |
|  | *Berberis thunbergii* DC. | 0.118 | 0.140 | 0.129 | AN, CA, CH(DE), CR(DA), SE, GF, GM(DE), MA(DE), MO(DA), MP, RB(DE), WP |
|  | *Cardamine impatiens* L. | 0.009 | 0.004 | 0.007 | VJ |
|  | *Celastrus orbiculatus* Thunb. | 0.100 | 0.096 | 0.098 | CH, CR, SE(DA), GF, GM(DE), MA, MO, MP, VJ, WP |
|  | *Cirsium arvense* (L.) Scop. | 0.031 | 0.022 | 0.026 | SE(DA) |
|  | *Commelina comm*unis L. | 0.009 | 0.009 | 0.009 | AN |
|  | *Dactylis glomerata* L. | 0.022 | 0.022 | 0.022 | RB(DE) |
|  | *Duchesnea indica* (Andrews) Focke | 0.140 | 0.140 | 0.140 | AN, CH, SE, GF(DE), MA, VJ(DA) |
|  | *Elaeagnus angustifolia* L. | 0.004 | 0 | 0.002 | AN(DA) |
|  | *Elaeagnus umbellata* Thunb. | 0.035 | 0.031 | 0.033 | AN(DA), CH(DE), CR, MA |
|  | *Epipactis helleborine* (L.) Crantz | 0 | 0.004 | 0.002 | WP(DE) |
|  | *Euonymus alatus* (Thunb.) Siebold | 0.004 | 0.009 | 0.007 | VJ |
|  | *Galium mollugo* L. | 0.026 | 0.026 | 0.026 | GF(DA) |
|  | *Glechoma hederacea* L. | 0.079 | 0.079 | 0.079 | AN, CH, CR(DA), SE, MA(DE), RB(DE), SH(DE) |
|  | *Hedera helix* L. | 0.004 | 0.009 | 0.007 | SE(DE), RB |
|  | *Hesperis matronalis* L. | 0.004 | 0.009 | 0.007 | CH |
|  | *Hypericum perforatum* L. | 0.017 | 0.013 | 0.015 | SE |
|  | *Lamium purpureum* L. | 0.009 | 0.017 | 0.013 | AN |
|  | *Leonurus cardiaca* L. | 0.009 | 0 | 0.004 | CR(DA), SH(DA) |
|  | *Ligustrum obtusifolium* Siebold & Zucc. | 0 | 0.004 | 0.002 | MP(DE) |
|  | *Ligustrum vulgare* L. | 0 | 0.017 | 0.009 | VJ(DE) |
|  | *Lonicera japonica* Thunb. | 0.297 | 0.384 | 0.341 | AN, CH, CR, SE, GF, GM(DE), MA, MO(DE), MP, R1(DE), RB, SH, VJ(DE) |
|  | *Lonicera maackii* (Rupr.) Herder | 0 | 0.031 | 0.015 | AN(DE), CH(DE), VJ(DE) |
|  | *Lonicera morrowii* A. Gray | 0 | 0.004 | 0.002 | VJ(DE) |
|  | *Lonicera xylosteum* L. | 0 | 0.004 | 0.002 | MP(DE) |
|  | *Microstegium vimineum* (Trin.) A. Camus | 0.376 | 0.271 | 0.323 | AN, CA, CH, CR, SE, GF, GM, MA, MO, MP(DA), RB, SH, VJ, WP |
|  | *Morus alba* L. | 0.004 | 0.031 | 0.017 | AN(DE), SE(DE), MO |
|  | *Paulownia tomentosa* (Thunb.) Siebold & Zucc. ex Steud. | 0 | 0.004 | 0.002 | SE(DE) |
|  | *Perilla frutescens* (L.) Britton | 0.079 | 0.035 | 0.057 | AN, CA(DE), CH(DA), CR, GM, MA(DA), MO(DA) |
|  | *Persicaria longiseta* (Bruijn) Kitag. | 0 | 0.009 | 0.004 | WP(DE) |
|  | *Plantago major* L. | 0 | 0.009 | 0.004 | SE(DE) |
|  | *Polygonum cespitosum* Blume, nom. inq. | 0.100 | 0.052 | 0.076 | CA, CH, CR, GF(DE), GM(DE), MA(DA), SH, VJ(DA), VM(DE) |
|  | *Polygonum convolvulus* L. | 0 | 0.004 | 0.002 | SH(DE) |
|  | *Polygonum perfoliatum* L. | 0.009 | 0.004 | 0.007 | CA, SE(DA) |
|  | *Polygonum persicaria* L. | 0.035 | 0.031 | 0.033 | CH, CR, SE(DA) |
|  | *Prunus avium* (L.) L. | 0.026 | 0.048 | 0.037 | AN(DE), CH, CR(DA), MA, MO(DE) |
|  | *Rhodotypos scanden*s (Thunb.) Makino | 0 | 0.004 | 0.002 | VJ(DE) |
|  | *Rosa multiflora* Thunb. | 0.148 | 0.227 | 0.188 | AN, CA(DE), CH, CR, SE, GF(DE), MA(DE), MO(DE), MP(DE), VJ(DE), WP |
|  | *Rubus phoenicolasius* Maxim. | 0.100 | 0.105 | 0.103 | AN, CA, CR, SE, GF(DA), GM, MA(DA), MO(DA), VJ(DE), WP |
|  | *Rumex obtusifolius* L. | 0.009 | 0.009 | 0.009 | CR(DA) |
|  | *Stellaria media* (L.) Vill. | 0.083 | 0.074 | 0.079 | AN, CR, MA(DA), MO, SH(DA) |
|  | *Trifolium campestre* Schreb. | 0 | 0.004 | 0.002 | SE(DE) |
|  | *Veronica hederifolia* L. | 0.009 | 0.004 | 0.007 | AN |
|  | *Veronica officinalis* L. | 0.013 | 0.004 | 0.009 | WP |
|  | *Vincetoxicum nigrum* (L.) Moench | 0 | 0.009 | 0.004 | WP(DE) |
| Native | *Acalypha rhomboidea* Raf. | 0.057 | 0.022 | 0.039 | CH, CR(DA) |
|  | *Acalypha virginica* L. | 0.026 | 0.026 | 0.026 | CR, MA, R1(DE) |
|  | *Acer negundo* L. | 0.192 | 0.188 | 0.190 | AN, CH, CR, SE, GF(DA), GM(DE), MO, SH(DA) |
|  | *Acer pensylvanicum* L. | 0.035 | 0.039 | 0.037 | MV, SH, WP |
|  | *Acer rubrum* L. | 0.437 | 0.463 | 0.450 | CA, CH, CR, SE, GF, GM, LR, MA, MO, MP(DA), MV, R1, RB(DE), SH, TR, VJ, VM, WP, Z1 |
|  | *Acer saccharum* Marsh. | 0.092 | 0.100 | 0.096 | AN(DE), CH, FE, LR, MP(DA), MV, SH(DA), TR, WP, Z1 |
|  | *Achillea millefolium* L. | 0.009 | 0.009 | 0.009 | WP(DA) |
|  | *Actaea pachypoda* Elliot | 0 | 0.004 | 0.002 | SH(DE) |
|  | *Actaea podocarpa* DC. | 0.004 | 0 | 0.002 | FN(DA) |
|  | *Actaea racemosa* L. var. racemosa | 0.039 | 0.074 | 0.057 | CA(DE), CR, GF(DE), R1, SH, TR(DE), VJ(DE), Z1 |
|  | *Adiantum pedatum* L. | 0.013 | 0.017 | 0.015 | CA(DA), GF(DA), R1, SH(DE), WP(DE), Z1(DE) |
|  | *Aesculus flava* Aiton | 0.004 | 0.004 | 0.004 | Z1 |
|  | *Ageratina altissima* (L.) King & H. Rob. var. altissima | 0.039 | 0.048 | 0.044 | FE, MP, SH(DE), TR, WP |
|  | *Agrimonia gryposepala* Wallr. | 0.013 | 0.017 | 0.015 | CH(DE), FE, MA |
|  | *Agrimonia parviflora* Aiton | 0.009 | 0.013 | 0.011 | CH(DA), SE |
|  | *Allium tricoccum* Aiton | 0.013 | 0.009 | 0.011 | FE |
|  | *Ambrosia artemisiifolia* L. | 0.035 | 0.017 | 0.026 | MO(DA) |
|  | *Amelanchier arborea* (Michx. f.) Fernald | 0.052 | 0.061 | 0.057 | SE(DE), GF, LR, MO(DE), MV, R1(DA), SH, Z1 |
|  | *Amianthium muscitoxicum* (Walter) A. Gray | 0 | 0.004 | 0.002 | SH(DE) |
|  | *Amphicarpaea bracteata* (L.) Fernald | 0.074 | 0.096 | 0.085 | CA(DA), CR, SE, FE, GF, MA, R1, SH, WP, Z1(DA) |
|  | *Anemone americana* (DC.) H. Hara | 0.009 | 0.009 | 0.009 | GF(DA), SH(DE), WP(DE), Z1(DA) |
|  | *Antennaria neglecta* Greene | 0.004 | 0 | 0.002 | WP(DA) |
|  | *Antennaria plantaginifolia* (L.) Richardson | 0.004 | 0 | 0.002 | SH(DA) |
|  | *Aquilegia canadensis* L. | 0.004 | 0 | 0.002 | SH(DA) |
|  | *Aralia nudicaulis* L. | 0 | 0.009 | 0.004 | VM(DE) |
|  | *Aralia spinosa* L. | 0.004 | 0.004 | 0.004 | MV |
|  | *Arisaema atrorubens* (Aiton) Blume var. stewardsonii (Britton) G.T. Stevens | 0.013 | 0.013 | 0.013 | TR |
|  | *Arisaema dracontium* (L.) Schott | 0 | 0.004 | 0.002 | Z1(DE) |
|  | *Arisaema triphyllum* (L.) Schott | 0.162 | 0.205 | 0.183 | AN(DA), CH, CR, SE, FE(DE), GF, GM, MG(DE), MP, R1(DE), SH, VJ, WP, Z1(DA) |
|  | *Aristolochia macrophylla* Lam. | 0 | 0.004 | 0.002 | SH(DE) |
|  | *Aristolochia serpentaria* L. | 0.013 | 0.035 | 0.024 | CR(DE), GF(DE), MA, R1(DA), SH(DE), WP, Z1(DE) |
|  | *Arnoglossum atriplicifolium* (L.) H. Rob. | 0.004 | 0 | 0.002 | SH(DA) |
|  | *Asarum canadense* L. | 0.031 | 0.039 | 0.035 | AN(DE), CH, FE(DE), TR, Z1 |
|  | *Asclepias exaltata* L. | 0 | 0.004 | 0.002 | SH(DE) |
|  | *Asimina triloba* (L.) Dunal | 0.122 | 0.100 | 0.111 | AN, CH, CR(DA), GF, GM, MA(DA), MO(DA), RB(DA), SH(DA) |
|  | *Asplenium platyneuron* (L.) Britton, Sterns & Poggenb. var. bacculum-rubrum (Featherm.) Fernald | 0.004 | 0.017 | 0.011 | SE(DE), MA |
|  | *Aster shortii* Lindl. | 0.009 | 0.009 | 0.009 | FE |
|  | *Athyrium filix-femina* (L.) Roth | 0.009 | 0.017 | 0.013 | SE(DE), GM, MG |
|  | *Aureolaria laevigata* (Raf.) Raf. | 0 | 0.004 | 0.002 | R1(DE) |
|  | *Betula alleghaniensis* Britton | 0.013 | 0 | 0.007 | CA(DA), MP(DA), SH(DA) |
|  | *Betula lenta* L. | 0.035 | 0.031 | 0.033 | CA, LR, MP, MV, VJ(DE) |
|  | *Betula papyrifera* Marsh. | 0.004 | 0 | 0.002 | MP(DA) |
|  | *Boehmeria cylindrica* (L.) Sw. | 0.070 | 0.096 | 0.083 | AN, CA(DE), CH(DA), CR(DE), SE, MA, SH(DE), VJ(DA) |
|  | *Botrychium dissectum* Spreng. | 0.004 | 0.009 | 0.007 | SE |
|  | *Botrychium virginianum* (L.) Sw. | 0.070 | 0.083 | 0.076 | AN(DE), CA(DE), CH, CR, SE, FE(DE), GM(DE), MA, RB(DE), SH, Z1(DA) |
|  | *Brachyelytrum erectum* (Schreb. ex Spreng.) P. Beauv. | 0.017 | 0.004 | 0.011 | R1(DA), WP, Z1(DA) |
|  | *Campanulastrum americanum* (L.) Small | 0.004 | 0 | 0.002 | FE(DA) |
|  | *Campsis radicans* (L.) Seem. ex Bureau | 0.013 | 0.013 | 0.013 | CH(DE), SE |
|  | *Cardamine concatenata* (Michx.) Sw. | 0.004 | 0.009 | 0.007 | AN(DE), TR |
|  | *Cardamine diphylla* (Michx.) Alph. Wood | 0.004 | 0.009 | 0.007 | MG(DE), TR |
|  | *Cardamine parviflora* L. | 0.009 | 0.004 | 0.007 | AN |
|  | *Cardamine pensylvanica* Muhl. ex Willd. | 0.004 | 0 | 0.002 | MP(DA) |
|  | *Carex albursina* Sheldon | 0.004 | 0 | 0.002 | FE(DA) |
|  | *Carex annectens* (E.P. Bicknell) E.P. Bicknell | 0.009 | 0.009 | 0.009 | SE |
|  | *Carex appalachica* J. Webber & P.W. Ball | 0.009 | 0 | 0.004 | WP(DA) |
|  | *Carex blanda* Dewey | 0.013 | 0.009 | 0.011 | FE, WP(DA) |
|  | *Carex digitalis* Willd. | 0.004 | 0.004 | 0.004 | R1(DE), WP(DA) |
|  | *Carex gracilescens* Steud. | 0.017 | 0.009 | 0.013 | R1, Z1 |
|  | *Carex grayi* Carey | 0.013 | 0.013 | 0.013 | MA |
|  | *Carex jamesii* Schwein. | 0.004 | 0.009 | 0.007 | FE |
|  | *Carex laxiculmis* Schwein. | 0.013 | 0.009 | 0.011 | FN(DA), WP |
|  | *Carex laxiflora* Lam. | 0.009 | 0.009 | 0.009 | WP |
|  | *Carex lurida* Wahlenb. | 0.004 | 0 | 0.002 | SE(DA) |
|  | *Carex pensylvanica* Lam. | 0.022 | 0.017 | 0.020 | VM(DA), WP |
|  | *Carex platyphylla* Carey | 0 | 0.004 | 0.002 | SH(DE) |
|  | *Carex radiata* (Wahlenb.) Small | 0.017 | 0.017 | 0.017 | MA, WP(DE) |
|  | *Carex rosea* Schkuhr ex Willd. | 0.004 | 0.013 | 0.009 | WP |
|  | *Carex squarrosa* L. | 0.004 | 0.004 | 0.004 | MA |
|  | *Carex swanii* (Fernald) Mack. | 0.017 | 0.035 | 0.026 | SE(DA), VJ(DE), WP |
|  | *Carex umbellata* Schkuhr ex Willd. | 0.004 | 0 | 0.002 | FN(DA) |
|  | *Carex virescens* Muhl. ex Willd. | 0.004 | 0 | 0.002 | WP(DA) |
|  | *Carex willdenowii* Schkuhr ex Willd. | 0.004 | 0 | 0.002 | R1(DA) |
|  | *Carpinus caroliniana* Walter | 0.118 | 0.118 | 0.118 | CA(DE), CH, SE, GF, GM, MA, MO, R1(DA), RB, SH, Z1 |
|  | *Carya alba* (L.) Nutt. | 0.044 | 0.092 | 0.068 | AN(DE), CH, SE, GF, MA(DE), MO(DE), R1, SH, VM(DE), Z1(DE) |
|  | *Carya cordiformis* (Wangenh.) K. Koch | 0.066 | 0.140 | 0.103 | AN(DE), CA, CH, CR, SE, FE, GF(DE), MA(DE), MO(DE), R1, SH(DE), WP, Z1(DA) |
|  | *Carya glabra* (Mill.) Sweet | 0.070 | 0.100 | 0.085 | CA, CH, CR(DA), SE, GF(DA), MA, MO, R1, SH, VJ(DE), Z1 |
|  | *Castanea dentata* (Marsh.) Borkh. | 0.004 | 0.013 | 0.009 | GF(DA), R1(DE), SH(DE), Z1(DE) |
|  | *Caulophyllum thalictroides* (L.) Michx. | 0.013 | 0.013 | 0.013 | CH, R1(DA), Z1(DE) |
|  | *Celtis occidentalis* L. | 0.162 | 0.197 | 0.179 | AN, CA(DE), CH, CR, FE, MA, MO, SH(DA) |
|  | *Cercis canadensis* L. | 0.057 | 0.079 | 0.068 | AN(DE), CR, GM(DE), MA, SH, VJ(DE) |
|  | *Chenopodium album* L. | 0.022 | 0.017 | 0.020 | FE(DA) |
|  | *Chimaphila maculata* (L.) Pursh | 0.044 | 0.048 | 0.046 | GF, MA(DA), R1, SH, VM, WP |
|  | *Chimaphila umbellata* (L.) W. Bartram | 0.017 | 0.009 | 0.013 | SE |
|  | *Cinna arundinacea* L. | 0.026 | 0.004 | 0.015 | SE, MA(DA) |
|  | *Circaea alpina* L. | 0 | 0.026 | 0.013 | VJ(DE) |
|  | *Circaea lutetiana* L. | 0.153 | 0.205 | 0.179 | AN, CA, CH, CR, SE, FE, GF, GM(DE), MA, MO, R1(DE), RB(DE), SH, TR, WP, Z1(DA) |
|  | *Claytonia virginica* L. | 0.004 | 0.017 | 0.011 | CH, VJ(DE) |
|  | *Clintonia borealis* (Aiton) Raf. | 0 | 0.004 | 0.002 | MG(DE) |
|  | *Collinsonia canadensis* L. | 0.009 | 0.004 | 0.007 | MG(DE), R1(DA) |
|  | *Conopholis americana* (L.) Wallr. | 0.009 | 0.017 | 0.013 | FN(DA), GF(DE), MG(DE), R1(DA), SH(DE), Z1(DE) |
|  | *Cornus florida* L. | 0.105 | 0.105 | 0.105 | AN, CR, SE, GF, GM(DE), MA, R1, RB(DA), SH, Z1 |
|  | *Corylus americana* Walter | 0 | 0.004 | 0.002 | R1(DE) |
|  | *Cryptotaenia canadensis* (L.) DC. | 0.004 | 0.004 | 0.004 | FE |
|  | *Cunila origanoides* (L.) Britton | 0.004 | 0 | 0.002 | R1(DA) |
|  | *Cynoglossum virginianum* L. | 0.013 | 0.013 | 0.013 | CR(DE), SE, SH(DA) |
|  | *Danthonia compressa* Austin | 0 | 0.004 | 0.002 | MG(DE) |
|  | *Danthonia spicata* (L.) P. Beauv. ex Roem. & Schult. | 0.004 | 0.009 | 0.007 | R1(DE), Z1 |
|  | *Dennstaedtia punctilobula* (Michx.) T. Moore | 0.070 | 0.096 | 0.083 | CA, SE(DE), GF(DE), GM(DE), LR, MV, VM, WP |
|  | *Desmodium glabellum* (Michx.) DC. | 0 | 0.009 | 0.004 | GF(DE), RB(DE) |
|  | *Desmodium nudiflorum* (L.) DC. | 0.026 | 0.031 | 0.028 | SE(DA), GF(DE), R1, VM(DE), Z1 |
|  | *Desmodium rotundifolium* DC. | 0.004 | 0.004 | 0.004 | WP |
|  | *Diarrhena americana* P. Beauv. | 0.009 | 0 | 0.004 | R1(DA) |
|  | *Dichanthelium acuminatum* (Sw.) Gould & C.A. Clark | 0.004 | 0.009 | 0.007 | WP |
|  | *Dichanthelium boscii* (Poir.) Gould & C.A. Clark | 0.039 | 0.026 | 0.033 | MA(DA), R1, WP, Z1(DA) |
|  | *Dichanthelium clandestinum* (L.) Gould | 0.004 | 0.009 | 0.007 | CR, FN(DE) |
|  | *Dichanthelium commutatum* (Schult.) Gould | 0.009 | 0.009 | 0.009 | SE(DE), R1 |
|  | *Dichanthelium dichotomum* (L.) Gould var. breve (Hitchc. & Chase) Gould & C.A. Clark | 0.013 | 0.013 | 0.013 | R1, Z1 |
|  | *Dichanthelium latifolium* (L.) Gould & C.A. Clark | 0.004 | 0 | 0.002 | Z1(DA) |
|  | *Dioscorea quaternata* J.F. Gmel. | 0.017 | 0.017 | 0.017 | FN, MG |
|  | *Dioscorea villosa* L. | 0.044 | 0.057 | 0.050 | CA(DE), CR(DE), GF, GM(DE), MA(DA), R1, SH, Z1 |
|  | *Dryopteris intermedia* (Muhl. ex Willd.) A. Gray | 0.017 | 0.013 | 0.015 | LR, MV |
|  | *Dryopteris marginalis* (L.) A. Gray | 0.004 | 0 | 0.002 | CA(DA) |
|  | *Dryopteris spinulosa* (O.F. Müll.) Watt | 0.004 | 0 | 0.002 | MG(DA) |
|  | *Dulichium arundinaceum* (L.) Britton | 0.004 | 0.004 | 0.004 | MA |
|  | *Eleocharis intermedia* Schult. | 0.017 | 0.017 | 0.017 | SE |
|  | *Elymus hystrix* L. | 0.009 | 0.013 | 0.011 | MA |
|  | *Elymus villosus* Muhl. ex Willd. | 0.022 | 0.026 | 0.024 | SE |
|  | *Elymus virginicus* L. | 0.017 | 0.022 | 0.020 | SE(DA), MA |
|  | *Epifagus virginiana* (L.) W. Bartram | 0 | 0.004 | 0.002 | SE(DE) |
|  | *Erechtites hieraciifolia* (L.) Raf. ex DC. | 0.026 | 0.013 | 0.020 | CH(DE), MA(DA), MO(DA), R1, Z1(DA) |
|  | *Erigeron annuus* (L.) Pers. | 0.026 | 0.031 | 0.028 | SE(DE) |
|  | *Erythronium americanum* Ker Gawl. | 0.004 | 0.004 | 0.004 | CR |
|  | *Euonymus americanus* L. | 0.009 | 0.004 | 0.007 | GF(DA), MA(DE), MO(DA) |
|  | *Eupatoriadelphus dubius* (Willd. ex Poir.) King & H. Rob. | 0.004 | 0 | 0.002 | SE(DA) |
|  | *Eupatorium purpureum* L. | 0.009 | 0.004 | 0.007 | FE(DA), VJ(DE) |
|  | *Eurybia divaricata* (L.) G.L. Nesom | 0.044 | 0.044 | 0.044 | CH, CR(DA), FN(DA), MG, TR(DE), WP |
|  | *Eurybia macrophylla* (L.) Cass. | 0.004 | 0.009 | 0.007 | MG(DE), R1 |
|  | *Euthamia graminifolia* (L.) Nutt. | 0.026 | 0.052 | 0.039 | SE(DE) |
|  | *Fagus grandifolia* Ehrh. | 0.083 | 0.096 | 0.090 | CA(DE), CH(DE), SE, GF(DA), MO, R1, WP, Z1 |
|  | *Fragaria* *virginiana* Duchesne | 0.004 | 0.013 | 0.009 | SE(DE), SH(DE) |
|  | *Fraxinus americana* L. | 0.183 | 0.253 | 0.218 | AN, CA, CH, CR, GF(DA), GM, MA, MO(DE), MP(DE), MV(DE), RB(DE), SH, TR, VJ(DE), Z1 |
|  | *Fraxinus pennsylvanica* Marsh. | 0.031 | 0.026 | 0.028 | SE |
|  | *Galearis spectabilis* (L.) Raf. | 0.009 | 0.013 | 0.011 | CR, GM(DE), SH |
|  | *Galium aparine* L. | 0.061 | 0.079 | 0.070 | AN, CA(DE), CR, SE, GF(DA), RB(DE), SH, TR |
|  | *Galium asprellum* Michx. | 0.013 | 0.004 | 0.009 | CH(DA), CR |
|  | *Galium circaezans* Michx. | 0.135 | 0.148 | 0.142 | AN(DE), CA, CR, SE, GF, GM(DE), MA(DA), R1, RB(DE), SH, VJ(DE), WP, Z1(DE) |
|  | *Galium concinnum* Torr. & A. Gray | 0.004 | 0.009 | 0.007 | FE, GM(DE) |
|  | *Galium lanceolatum* Torr. | 0.022 | 0.035 | 0.028 | CH(DE), CR, SH(DE), Z1 |
|  | *Galium latifolium* Michx. | 0.009 | 0.004 | 0.007 | SH |
|  | *Galium pilosum* Aiton | 0.004 | 0.004 | 0.004 | MA |
|  | *Galium triflorum* Michx. | 0.118 | 0.118 | 0.118 | AN, CH(DA), CR, SE, GM(DE), MA, R1, SH, WP, Z1 |
|  | *Gaultheria procumbens* L. | 0.004 | 0 | 0.002 | MG(DA) |
|  | *Gaylussacia baccata* (Wangenh.) K. Koch | 0.009 | 0.022 | 0.015 | GF, VM(DE) |
|  | *Gaylussacia frondosa* (L.) Torr. & A. Gray ex Torr. | 0.009 | 0.022 | 0.015 | VM(DE), WP |
|  | *Geranium maculatum* L. | 0.044 | 0.057 | 0.050 | CR(DA), FE, R1, SH(DE), TR, WP, Z1 |
|  | *Geum canadense* Jacq. | 0.105 | 0.162 | 0.133 | CH, CR, SE, FE, GF(DA), MA(DE), RB(DE), SH, VJ(DE), WP(DE) |
|  | *Geum laciniatum* Murray | 0 | 0.009 | 0.004 | CH(DE), CR(DE) |
|  | *Geum virginianum* L. | 0 | 0.004 | 0.002 | WP(DE) |
|  | *Gleditsia triacanthos* L. | 0 | 0.004 | 0.002 | CH(DE) |
|  | *Goodyera pubescens* (Willd.) R. Br. | 0.004 | 0.004 | 0.004 | R1 |
|  | *Hackelia virginiana* (L.) I.M. Johnst. | 0.009 | 0.004 | 0.007 | FE |
|  | *Hamamelis virginiana* L. | 0.035 | 0.039 | 0.037 | R1, SH, VM, WP |
|  | *Hedeoma pulegioides* (L.) Pers. | 0.004 | 0 | 0.002 | CA(DA) |
|  | *Helianthus divaricatus* L. | 0 | 0.004 | 0.002 | SH(DE) |
|  | *Helianthus microcephalus* Torr. & A. Gray | 0 | 0.004 | 0.002 | R1(DE) |
|  | *Hepatica nobilis* Schreb. | 0 | 0.009 | 0.004 | MG(DE), VJ(DE) |
|  | *Heuchera pubescens* Pursh | 0.004 | 0 | 0.002 | MA(DA) |
|  | *Hieracium paniculatum* L. | 0.004 | 0.004 | 0.004 | WP |
|  | *Hieracium scabrum* Michx. | 0.004 | 0.004 | 0.004 | SE |
|  | *Hieracium venosum* L. | 0.004 | 0 | 0.002 | SH(DA) |
|  | *Houstonia caerulea* L. | 0.004 | 0.004 | 0.004 | R1 |
|  | *Houstonia longifolia* Gaertn. | 0.009 | 0 | 0.004 | SH(DA) |
|  | *Hydrangea arborescens* L. | 0.009 | 0.013 | 0.011 | R1(DE), Z1 |
|  | *Hydrophyllum appendiculatum* Michx. | 0.004 | 0 | 0.002 | FE(DA) |
|  | *Hydrophyllum virginianum* L. | 0.031 | 0.044 | 0.037 | CH, FE, SH, TR |
|  | *Hystrix patula* Moench | 0.009 | 0.009 | 0.009 | FE |
|  | *Ilex montana* Torr. & A. Gray ex A. Gray | 0.026 | 0.026 | 0.026 | LR, MV |
|  | *Ilex opaca* Aiton | 0.057 | 0.057 | 0.057 | SE, GF, GM, MA |
|  | *Impatiens capensis* Meerb. | 0.022 | 0.026 | 0.024 | CH, CR(DE), SH(DA), TR |
|  | *Impatiens pallida* Nutt. | 0.009 | 0.009 | 0.009 | FE |
|  | *Isotria verticillata* Raf. | 0 | 0.004 | 0.002 | VM(DE) |
|  | *Juglans nigra* L. | 0.009 | 0.022 | 0.015 | AN(DE), FE(DA), MP(DA) |
|  | *Juncus tenuis* Willd. | 0.022 | 0.017 | 0.020 | WP |
|  | *Juniperus virginiana* L. | 0.039 | 0.057 | 0.048 | AN, CH(DE), CR, SE, GF(DE), MA, SH(DE) |
|  | *Kalmia angustifolia* L. | 0 | 0.009 | 0.004 | VM(DE) |
|  | *Kalmia latifolia* L. | 0.013 | 0.039 | 0.026 | GF, SH, VM, WP(DE) |
|  | *Krigia biflora* (Walter) S.F. Blake | 0.004 | 0 | 0.002 | R1(DA) |
|  | *Laportea canadensis* (L.) Weddell | 0.022 | 0.044 | 0.033 | CH, FE, SH(DE), TR |
|  | *Leersia virginica* Willd. | 0.035 | 0.022 | 0.028 | CH(DA), FE(DA), MA, WP |
|  | *Lespedeza procumbens* Michx. | 0 | 0.004 | 0.002 | MA(DE) |
|  | *Lespedeza violacea* (L.) Pers. | 0 | 0.004 | 0.002 | WP(DE) |
|  | *Lindera benzoin* (L.) Blume | 0.306 | 0.389 | 0.347 | AN, CH, CR, SE, GF, GM, MA, MO(DE), MP, R1, RB, SH, TR, VJ, Z1 |
|  | *Liquidambar styraciflua* L. | 0.057 | 0.057 | 0.057 | SE |
|  | *Liriodendron tulipifera* L. | 0.332 | 0.310 | 0.321 | AN, CA, CR, SE, GF, GM, MA, MO, MP, R1, RB(DA), SH, VJ, VM, WP, Z1 |
|  | *Lobelia inflata* L. | 0.013 | 0.022 | 0.017 | WP(DE) |
|  | *Lycopodium clavatum* L. | 0.009 | 0.017 | 0.013 | LR, MV |
|  | *Lycopodium digitatum* Dill. ex A. Braun | 0.039 | 0.039 | 0.039 | LR, MV |
|  | *Lysimachia ciliata* L. | 0.004 | 0.009 | 0.007 | GM(DE), MA(DE), Z1(DA) |
|  | *Lysimachia quadriflora* Sims | 0.009 | 0.004 | 0.007 | R1(DA), Z1 |
|  | *Lysimachia quadrifolia* L. | 0.009 | 0.013 | 0.011 | WP |
|  | *Magnolia acuminata* (L.) L. | 0.022 | 0.017 | 0.020 | MV |
|  | *Maianthemum canadense* Desf. | 0.087 | 0.070 | 0.079 | AN(DA), GF(DA), LR, MO(DE), MP, MV, VM, WP |
|  | *Maianthemum racemosum* (L.) Link | 0.087 | 0.179 | 0.133 | CA, CH, CR, SE, FE, GF, LR(DE), MA, MP(DE), R1, SH(DE), TR, VJ(DE), VM(DE), WP, Z1 |
|  | *Medeola virginiana* L. | 0.044 | 0.066 | 0.055 | CA, FN(DE), GF, LR, MG, MV, SH(DE), VM(DE) |
|  | *Menispermum canadense* L. | 0.013 | 0.009 | 0.011 | AN(DA), CR(DE), FE, SH(DA) |
|  | *Mikania scandens* (L.) Willd. | 0 | 0.004 | 0.002 | SE(DE) |
|  | *Milium effusum* L. | 0.004 | 0.004 | 0.004 | WP |
|  | *Mitchella repens* L. | 0.100 | 0.083 | 0.092 | SE, FN(DE), GF, GM, LR, MA, MP, MV, R1(DA), WP |
|  | *Mitella diphylla* L. | 0.004 | 0 | 0.002 | GF(DA) |
|  | *Monarda clinopodia* L. | 0 | 0.004 | 0.002 | SH(DE) |
|  | *Monotropa uniflora* L. | 0 | 0.004 | 0.002 | SH(DE) |
|  | *Morus rubra* L. | 0.004 | 0.004 | 0.004 | MA(DE), R1(DA) |
|  | *Muhlenbergia schreberi* J.F. Gmel. | 0.039 | 0.013 | 0.026 | SE, MA(DA) |
|  | *Muhlenbergia tenuiflora* (Willd.) Britton, Sterns & Poggenb. | 0.004 | 0 | 0.002 | R1(DA) |
|  | *Nyssa sylvatica* Marsh. | 0.144 | 0.131 | 0.138 | CA(DA), CR(DA), SE(DE), GF, MA(DE), MO, R1, RB(DA), SH, VJ, VM, WP, Z1(DA) |
|  | *Obolaria virginica* L. | 0 | 0.004 | 0.002 | GF(DE) |
|  | *Onoclea sensibilis* L. | 0.004 | 0.009 | 0.007 | CR(DE), GM(DE) |
|  | *Ophioglossum vulgatum* L. | 0.004 | 0 | 0.002 | SE(DA) |
|  | *Osmorhiza claytonii* (Michx.) C.B. Clarke | 0.026 | 0.057 | 0.041 | AN(DE), CA(DE), CR(DE), FE, GF(DE), R1(DA), SH, VJ(DE), Z1 |
|  | *Osmorhiza longistylis* (Torr.) DC. | 0.022 | 0.017 | 0.020 | SE |
|  | *Osmunda cinnamomea* L. | 0.004 | 0.009 | 0.007 | GM(DA), LR(DE), MG(DE) |
|  | *Osmunda claytoniana* L. | 0.009 | 0.013 | 0.011 | WP, Z1 |
|  | *Ostrya virginiana* (Mill.) K. Koch | 0.026 | 0.031 | 0.028 | CH(DE), FE, GF(DA), GM(DE), RB(DA), SH, Z1 |
|  | *Oxalis europaea* Jord. | 0.105 | 0.079 | 0.092 | CH, SE, FE, GM(DE), MA, SH, WP |
|  | *Oxalis montana* Raf. | 0.031 | 0.026 | 0.028 | LR, MV |
|  | *Oxalis violacea* L. | 0.017 | 0 | 0.009 | CH(DA), GF(DA) |
|  | *Oxydendrum arboreum* (L.) DC. | 0.009 | 0.013 | 0.011 | R1, Z1 |
|  | *Panax quinquefolius* L. | 0 | 0.009 | 0.004 | CA(DE), Z1(DE) |
|  | *Parietaria pensylvanica* Muhl. ex Willd. | 0.013 | 0.004 | 0.009 | SH(DA), WP(DE) |
|  | *Paronychia canadensis* (L.) Alph. Wood | 0.013 | 0.013 | 0.013 | CA(DA), CR(DE), MA, SH(DA) |
|  | *Parthenocissus quinquefolia* (L.) Planch. | 0.515 | 0.581 | 0.548 | AN, CA, CH, CR, SE, FE, GF, GM, MA, MG(DE), MO, MP(DE), R1, RB, SH, VJ, VM, WP, Z1 |
|  | *Phaseolus* *polystachios* (L.) Britton, Sterns & Poggenb. | 0 | 0.004 | 0.002 | AN(DE) |
|  | *Phegopteris hexagonoptera* (Michx.) Fée | 0.013 | 0.004 | 0.009 | GF(DA), Z1 |
|  | *Phlox divaricata* L. | 0.009 | 0.004 | 0.007 | FE |
|  | *Phryma leptostachya* L. | 0.070 | 0.066 | 0.068 | AN, CR, SE, FE, GF(DA), GM(DE), MA |
|  | *Phytolacca americana* L. | 0.070 | 0.074 | 0.072 | CA, CH(DA), SE(DE), MO, TR(DA), VJ(DE) |
|  | *Pilea pumila* (L.) A. Gray | 0.114 | 0.061 | 0.087 | AN, CA(DA), CH, CR, FE, MA(DA), R1(DE), SH, WP |
|  | *Pinus virginiana* Mill. | 0.013 | 0 | 0.007 | MA(DA), SH(DA) |
|  | *Platanus occidentalis* L. | 0.004 | 0.004 | 0.004 | SE |
|  | *Poa cuspidata* Nutt. | 0.004 | 0.004 | 0.004 | R1 |
|  | *Podophyllum peltatum* L. | 0.044 | 0.066 | 0.055 | AN, CH(DA), SE, FE, SH(DE), TR, VJ(DE), Z1 |
|  | *Polemonium reptans* L. | 0.004 | 0.004 | 0.004 | Z1 |
|  | *Polygonatum biflorum* (Walter) Elliot | 0.048 | 0.114 | 0.081 | AN(DE), CA(DE), SE, FE, FN(DA), MA, MO(DE), R1, SH(DE), TR, VJ(DE), VM(DE), Z1(DE) |
|  | *Polygonatum pubescens* (Willd.) Pursh | 0 | 0.017 | 0.009 | MG(DE), MV(DE), WP(DE) |
|  | *Polygonum pensylvanicum* L. | 0.026 | 0.026 | 0.026 | SE |
|  | *Polygonum sagittatum* L. | 0 | 0.004 | 0.002 | WP(DE) |
|  | *Polygonum virginianum* L. | 0.162 | 0.175 | 0.168 | AN, CA(DE), CH, CR, SE, FE, GF(DA), GM(DE), MA, R1, VJ(DE), Z1(DA) |
|  | *Polymnia uvedalia* (L.) L. | 0 | 0.004 | 0.002 | AN(DE) |
|  | *Polystichum acrostichoides* (Michx.) Schott | 0.118 | 0.140 | 0.129 | CA(DE), CH, SE, FN, GF, GM(DE), MA(DE), MG, R1, RB(DA), WP, Z1 |
|  | *Populus grandidentata* Michx. | 0.004 | 0 | 0.002 | SH(DA) |
|  | *Potentilla canadensis* L. | 0.022 | 0.013 | 0.017 | SE, SH, Z1(DA) |
|  | *Potentilla simplex* Michx. | 0.031 | 0.035 | 0.033 | R1, WP, Z1 |
|  | *Prenanthes alba* L. | 0 | 0.013 | 0.007 | SH(DE) |
|  | *Prenanthes altissima* L. | 0 | 0.009 | 0.004 | CA(DE), VM(DE) |
|  | *Prenanthes trifoliolata* (Cass.) Fernald | 0.004 | 0 | 0.002 | FN(DA) |
|  | *Prosartes lanuginosa* (Michx.) D. Don | 0.013 | 0.004 | 0.009 | FN, MG(DA) |
|  | *Prunus serotina* Ehrh. | 0.306 | 0.415 | 0.360 | AN, CA(DE), CH, CR, SE, FE, GF, GM, LR, MA, MO, MV, RB(DE), SH, TR, VJ(DE), VM, WP, Z1 |
|  | *Prunus virginiana* L. | 0.004 | 0.004 | 0.004 | FE |
|  | *Pteridium aquilinum* (L.) Kuhn | 0.009 | 0.013 | 0.011 | MV(DE), SH(DA), VJ(DE), VM(DE) |
|  | *Pycnanthemum incanum* (L.) Michx. | 0.004 | 0 | 0.002 | SH(DA) |
|  | *Pycnanthemum tenuifolium* Schrad. | 0 | 0.004 | 0.002 | MA(DE) |
|  | *Pyrola americana* Sweet | 0.004 | 0.004 | 0.004 | WP |
|  | *Quercus alba* L. | 0.044 | 0.070 | 0.057 | FE(DE), GF, GM(DE), MA, R1, RB(DE), SH, WP, Z1 |
|  | *Quercus coccinea* Münchh. | 0.009 | 0.013 | 0.011 | R1, Z1 |
|  | *Quercus falcata* Michx. | 0.004 | 0.004 | 0.004 | SE |
|  | *Quercus marilandica* Münchh. | 0.004 | 0 | 0.002 | SE(DA) |
|  | *Quercus palustris* Münchh. | 0.004 | 0 | 0.002 | SE(DA) |
|  | *Quercus prinus* L. | 0.087 | 0.131 | 0.109 | CA, CR(DA), GF, MO, MP(DA), R1(DE), SH, VJ(DE), VM, WP(DE) |
|  | *Quercus rubra* L. | 0.096 | 0.153 | 0.124 | CH(DA), SE(DA), FE, GF, GM(DA), MA(DE), R1, SH, VJ(DE), VM(DE), WP, Z1 |
|  | *Quercus stellata* Wangenh. | 0 | 0.009 | 0.004 | MA(DE) |
|  | *Quercus velutina* Lam. | 0.044 | 0.057 | 0.050 | SE, MO, R1, SH, VJ(DE), VM(DE), Z1 |
|  | *Ranunculus abortivus* L. | 0.035 | 0.031 | 0.033 | AN, FE(DA), SH |
|  | *Ranunculus hispidus* Michx. | 0.004 | 0 | 0.002 | R1(DA) |
|  | *Ranunculus septentrionalis* Poir. | 0.004 | 0.004 | 0.004 | FE |
|  | *Rhododendron periclymenoides* (Michx.) Shinners | 0.004 | 0.039 | 0.022 | GF(DE), SH, VJ(DE), VM(DE) |
|  | *Rhododendron prinophyllum* (Small) Millais | 0.009 | 0.004 | 0.007 | WP |
|  | *Rhus copallinum* L. | 0 | 0.004 | 0.002 | MP(DE) |
|  | *Rhus glabra* L. | 0 | 0.004 | 0.002 | Z1(DE) |
|  | *Ribes missouriense* Nutt. | 0 | 0.004 | 0.002 | FE(DE) |
|  | *Robinia pseudoacacia* L. | 0.026 | 0.022 | 0.024 | AN, CA, CH(DE), SE(DA), SH |
|  | *Rosa carolina* L. | 0.009 | 0.009 | 0.009 | R1, Z1 |
|  | *Rubus allegheniensis* Porter | 0.074 | 0.100 | 0.087 | SE, FN(DA), LR, MO, MV, RB(DE) |
|  | *Rubus canadensis* L. | 0.004 | 0 | 0.002 | MA(DA) |
|  | *Rubus flagellaris* Willd. | 0.004 | 0.026 | 0.015 | MA, WP(DE) |
|  | *Rubus occidentalis* L. | 0.013 | 0.039 | 0.026 | AN(DE), CH(DE), CR, SE(DA), FE(DE), MO(DE) |
|  | *Rubus pensilvanicus* Poir. | 0 | 0.026 | 0.013 | AN(DE), VJ(DE), VM(DE) |
|  | *Rudbeckia hirta* L. var. pulcherrima Farw. | 0 | 0.004 | 0.002 | MA(DE) |
|  | *Ruellia strepens* L. | 0.035 | 0.004 | 0.020 | CH(DA), CR, MA(DA) |
|  | *Sambucus canadensis* L. | 0 | 0.013 | 0.007 | CA(DE), SE(DE), SH(DE) |
|  | *Sanguinaria canadensis* L. | 0.017 | 0.035 | 0.026 | AN(DA), CA(DA), CR(DA), FE(DE), GF(DE), VJ(DE) |
|  | *Sanicula canadensis* L. | 0.070 | 0.061 | 0.066 | AN(DE), CR(DA), SE, MG(DE), SH(DE) |
|  | *Sanicula gregaria* E.P. Bicknell | 0.017 | 0.031 | 0.024 | AN, FE, GF(DE), MO |
|  | *Sanicula marilandica* L. | 0.004 | 0.004 | 0.004 | TR |
|  | *Sassafras albidum* (Nutt.) Nees | 0.140 | 0.201 | 0.170 | CA, CH, CR, SE, FE(DA), GF, GM(DE), MA(DA), MO(DE), MP(DE), R1, SH, VJ, VM, WP, Z1 |
|  | *Saururus cernuus* L. | 0.004 | 0.004 | 0.004 | MA |
|  | *Scutellaria lateriflora* L. | 0.004 | 0 | 0.002 | WP(DA) |
|  | *Sedum ternatum* Michx. | 0.009 | 0.009 | 0.009 | CH(DA), MA(DA), SH(DE), TR(DE) |
|  | *Smilax ecirrhata* (Engelm. ex Kunth) S. Watson | 0.013 | 0.013 | 0.013 | FE |
|  | *Smilax glauca* Walter | 0.118 | 0.122 | 0.120 | CH, CR(DA), GF, GM, MA, R1, VJ, VM, Z1 |
|  | *Smilax herbacea* L. | 0 | 0.004 | 0.002 | CR(DE) |
|  | *Smilax rotundifolia* L. | 0.157 | 0.131 | 0.144 | CA, CH(DA), CR, SE, FN, GF, GM, MA, MG, MO(DE), R1, Z1 |
|  | *Smilax tamnoides* L. | 0.017 | 0.044 | 0.031 | AN(DE), CH, GF, MA(DE) |
|  | *Solidago altissima* L. | 0.048 | 0.052 | 0.050 | SE, FE(DA) |
|  | *Solidago caesia* L. | 0.026 | 0.035 | 0.031 | CR(DE), SE(DA), GF(DE), R1, WP |
|  | *Solidago flexicaulis* L. | 0.004 | 0.009 | 0.007 | FE(DA), GM(DE), SH(DE) |
|  | *Solidago rugosa* Mill. | 0.035 | 0.039 | 0.037 | SE |
|  | *Stellaria pubera* Michx. | 0.017 | 0.017 | 0.017 | SH |
|  | *Streptopus lanceolatus* (Aiton) Reveal var. roseus (Michx.) Reveal | 0.013 | 0.009 | 0.011 | MG |
|  | *Symphoricarpos orbiculatus* Moench | 0.048 | 0.026 | 0.037 | CR, SH |
|  | *Symphyotrichum cordifolium* (L.) G.L. Nesom | 0.004 | 0 | 0.002 | R1(DA) |
|  | *Symphyotrichum divaricatum* (Nutt.) G.L. Nesom | 0.017 | 0.022 | 0.020 | R1, VJ(DE), VM(DE), Z1 |
|  | *Symphyotrichum lateriflorum* (L.) A. Löve & D. Löve | 0.039 | 0.048 | 0.044 | WP |
|  | *Symphyotrichum lateriflorum* (L.) A. Löve & D. Löve var. lateriflorum | 0.009 | 0 | 0.004 | SE(DA) |
|  | *Symphyotrichum undulatum* (L.) G.L. Nesom | 0 | 0.004 | 0.002 | R1(DE) |
|  | *Taraxacum officinale* F.H. Wigg. | 0.035 | 0.031 | 0.033 | CR(DE), SE(DA), MO(DA) |
|  | *Teucrium canadense* L. | 0 | 0.004 | 0.002 | CR(DE) |
|  | *Thalictrum coriaceum* (Britton) Small | 0.009 | 0.013 | 0.011 | SH |
|  | *Thalictrum dioicum* L. | 0 | 0.004 | 0.002 | WP(DE) |
|  | *Thalictrum thalictroides* (L.) Eames & B. Boivin | 0.039 | 0.031 | 0.035 | CA, CR, GF(DA), MG(DA), R1, SH, Z1 |
|  | *Thelypteris noveboracensis* (L.) Nieuwl. | 0.031 | 0.044 | 0.037 | GF, GM, LR(DE), MG, MV, VM, Z1(DA) |
|  | *Tiarella cordifolia* L. | 0 | 0.004 | 0.002 | R1(DE) |
|  | *Tilia americana* L. | 0.017 | 0.026 | 0.022 | CA(DE), FE, SH(DE), WP |
|  | *Toxicodendron radicans* (L.) Kuntze | 0.306 | 0.393 | 0.349 | AN, CA, CH, CR, SE, FE, GF, GM(DE), MA, MO(DE), MP(DE), R1, RB(DE), SH, VJ, VM, WP, Z1 |
|  | *Trientalis borealis* Raf. | 0.044 | 0.044 | 0.044 | LR, MV, WP |
|  | *Trillium erectum* L. | 0.004 | 0 | 0.002 | TR(DA) |
|  | *Trillium grandiflorum* (Michx.) Salisb. | 0.013 | 0.017 | 0.015 | FE(DE), TR, Z1(DE) |
|  | *Trillium recurvatum* Beck | 0.004 | 0.009 | 0.007 | FE |
|  | *Trillium undulatum* Willd. | 0 | 0.004 | 0.002 | MV(DE) |
|  | *Ulmus americana* L. | 0.013 | 0.044 | 0.028 | CH, CR |
|  | *Ulmus rubra* Muhl. | 0.079 | 0.166 | 0.122 | AN, CH, CR(DE), SE, GF(DE), MA, MO, SH, VJ(DE) |
|  | *Urtica dioica* L. | 0.035 | 0.013 | 0.024 | AN, CH |
|  | *Uvularia grandiflora* Sm. | 0.009 | 0.013 | 0.011 | SE(DE), FE |
|  | *Uvularia perfoliata* L. | 0.048 | 0.096 | 0.072 | CA, SE, GF(DE), GM(DE), MA(DE), R1, SH, VJ(DE), VM(DE), WP(DE), Z1 |
|  | *Uvularia sessilifolia* L. | 0.044 | 0.035 | 0.039 | GF(DE), LR, RB, SH, WP |
|  | *Vaccinium angustifolium* Aiton | 0.031 | 0.052 | 0.041 | VJ(DE), VM, WP |
|  | *Vaccinium corymbosum* L. | 0 | 0.009 | 0.004 | SE(DE), MP(DE) |
|  | *Vaccinium fuscatum* Aiton | 0 | 0.004 | 0.002 | MP(DE) |
|  | *Vaccinium pallidum* Aiton | 0.074 | 0.079 | 0.076 | GF, MA(DE), MP(DA), R1, SH(DA), WP, Z1 |
|  | *Vaccinium stamineum* L. | 0.017 | 0.022 | 0.020 | GF(DE), MA(DA), R1(DA), VM(DE), WP(DE), Z1 |
|  | *Veratrum viride* Aiton | 0.004 | 0 | 0.002 | SH(DA) |
|  | *Verbena urticifolia* L. | 0.017 | 0.009 | 0.013 | CR |
|  | *Verbesina alternifolia* (L.) Britton ex Kearney | 0.035 | 0.048 | 0.041 | CH, CR, MA(DE) |
|  | *Verbesina occidentalis* (L.) Walter | 0.013 | 0.017 | 0.015 | CH |
|  | *Viburnum acerifolium* L. | 0.039 | 0.122 | 0.081 | SE, GF, MA(DE), MP(DE), R1, SH(DE), VJ(DE), VM(DE), WP, Z1 |
|  | *Viburnum dentatum* L. | 0.013 | 0.035 | 0.024 | SE, GF(DE), MA(DE), RB(DE), VM(DE), WP(DE) |
|  | *Viburnum prunifolium* L. | 0.109 | 0.127 | 0.118 | AN(DE), CH, CR(DE), SE, MA, R1(DE), VJ, VM(DA), Z1(DA) |
|  | *Viola labradorica* Schrank | 0.004 | 0.009 | 0.007 | WP |
|  | *Viola palmata* L. | 0.009 | 0.009 | 0.009 | AN(DE), CR |
|  | *Viola papilionacea* Pursh p.p. | 0.100 | 0.083 | 0.092 | CA, SE, FE, SH(DA), TR(DE), WP |
|  | *Viola pensylvanica* Michx. | 0.048 | 0.044 | 0.046 | CH, FE, SH |
|  | *Viola striata* Aiton | 0.009 | 0.009 | 0.009 | CH |
|  | *Viola triloba* Schwein. | 0.031 | 0.035 | 0.033 | CR(DA), GF(DE), MA(DE), MG(DE), R1, SH, Z1(DA) |
|  | *Vitis aestivalis* Michx. | 0.004 | 0.004 | 0.004 | VJ(DA), VM(DE) |
|  | *Vitis labrusca* L. | 0.022 | 0.017 | 0.020 | SH, WP(DE) |
|  | *Vitis riparia* Michx. | 0.013 | 0.009 | 0.011 | FE, WP |
|  | *Vitis vulpina* L. | 0.052 | 0.066 | 0.059 | SE, VJ(DE) |
| Unknown | *Acalypha* L. | 0.004 | 0.004 | 0.004 | AN |
|  | *Acalypha* L. | 0.004 | 0.004 | 0.004 | CH |
|  | *Acalypha* L. | 0 | 0.004 | 0.002 | GM(DE) |
|  | *Acalypha* L. | 0.004 | 0 | 0.002 | MO(DA) |
|  | *Acer* L. | 0.013 | 0.009 | 0.011 | CH |
|  | *Acer* L. | 0.004 | 0.004 | 0.004 | MP |
|  | *Agrimonia* L. | 0.004 | 0 | 0.002 | CH(DA) |
|  | *Agrimonia* L. | 0.004 | 0 | 0.002 | CR(DA) |
|  | *Agrimonia* L. | 0.004 | 0.004 | 0.004 | R1 |
|  | *Agrimonia* L. | 0.004 | 0 | 0.002 | Z1(DA) |
|  | *Allium* L. | 0.004 | 0.009 | 0.007 | AN |
|  | *Allium* L. | 0.004 | 0.004 | 0.004 | VJ |
|  | *Alysicarpus* Neck. ex Desv. | 0.004 | 0 | 0.002 | CH(DA) |
|  | *Amelanchier* Medik. | 0.004 | 0 | 0.002 | SE(DA) |
|  | *Andropogon* L. | 0 | 0.009 | 0.004 | MA(DE) |
|  | *Antennaria* Gaertn. | 0 | 0.004 | 0.002 | MA(DE) |
|  | APF | 0.004 | 0 | 0.002 | SH(DA) |
|  | *Apiaceae* | 0 | 0.004 | 0.002 | AN(DE) |
|  | *Apiaceae* | 0.004 | 0.009 | 0.007 | CR |
|  | APL | 0.004 | 0 | 0.002 | SH(DA) |
|  | *Apocynum* L. | 0 | 0.004 | 0.002 | MA(DE) |
|  | APR | 0 | 0.004 | 0.002 | SH(DE) |
|  | *Aralia* L. | 0.004 | 0.004 | 0.004 | CH |
|  | *Arisaema* Mart. | 0.013 | 0.022 | 0.017 | CA |
|  | *Asclepias* L. | 0 | 0.004 | 0.002 | MP(DE) |
|  | *Asplenium* L. | 0.004 | 0.004 | 0.004 | AN |
|  | *Aster* L. | 0.013 | 0.004 | 0.009 | FE |
|  | *Aster* L. | 0 | 0.004 | 0.002 | MG(DE) |
|  | *Aster* L. | 0.004 | 0.013 | 0.009 | R1 |
|  | *Aster* L. | 0 | 0.009 | 0.004 | SH(DE) |
|  | *Aster* L. | 0.004 | 0 | 0.002 | TR(DA) |
|  | *Aster* L. | 0.004 | 0 | 0.002 | WP(DA) |
|  | *Aster* L. | 0.004 | 0 | 0.002 | Z1(DA) |
|  | *Asteraceae* | 0 | 0.004 | 0.002 | CR(DE) |
|  | *Asteraceae* | 0 | 0.004 | 0.002 | GF(DE) |
|  | *Asteraceae* | 0 | 0.004 | 0.002 | GM(DE) |
|  | *Asteraceae* | 0.004 | 0.013 | 0.009 | MA |
|  | *Asteraceae* | 0.004 | 0 | 0.002 | MO(DA) |
|  | *Asteraceae* | 0 | 0.004 | 0.002 | RB(DE) |
|  | *Asteraceae* | 0.004 | 0 | 0.002 | WP(DA) |
|  | ATC | 0.004 | 0 | 0.002 | SH(DA) |
|  | *Athyrium* Roth | 0 | 0.004 | 0.002 | GM(DE) |
|  | *Berberis* L. | 0 | 0.004 | 0.002 | CR(DE) |
|  | *Betula* L. | 0 | 0.004 | 0.002 | TR(DE) |
|  | *Betula* L. | 0.031 | 0.031 | 0.031 | WP |
|  | *Bidens* L. | 0.004 | 0 | 0.002 | FE(DA) |
|  | *Botrychium* Sw. | 0.004 | 0.004 | 0.004 | SE |
|  | C1 | 0 | 0.009 | 0.004 | CH(DE) |
|  | C2 | 0.004 | 0.004 | 0.004 | CH |
|  | C5 | 0.004 | 0.004 | 0.004 | CH |
|  | C6 | 0.009 | 0.004 | 0.007 | CH |
|  | C7 | 0.004 | 0 | 0.002 | CH(DA) |
|  | C8 | 0.004 | 0 | 0.002 | CH(DA) |
|  | C9 | 0.004 | 0 | 0.002 | CH(DA) |
|  | *Cardamine* L. | 0.009 | 0 | 0.004 | MP(DA) |
|  | *Carex* L. | 0.013 | 0.013 | 0.013 | AN |
|  | *Carex* L. | 0.017 | 0.009 | 0.013 | CA |
|  | *Carex* L. | 0.044 | 0.048 | 0.046 | CH |
|  | *Carex* L. | 0.031 | 0.022 | 0.026 | CR |
|  | *Carex* L. | 0.031 | 0.026 | 0.028 | SE |
|  | *Carex* L. | 0.017 | 0.026 | 0.022 | GF |
|  | *Carex* L. | 0.022 | 0.026 | 0.024 | GM |
|  | *Carex* L. | 0.004 | 0 | 0.002 | LR(DA) |
|  | *Carex* L. | 0.057 | 0.031 | 0.044 | MA |
|  | *Carex* L. | 0.004 | 0 | 0.002 | MG(DA) |
|  | *Carex* L. | 0.009 | 0.009 | 0.009 | MO |
|  | *Carex* L. | 0.004 | 0 | 0.002 | MP(DA) |
|  | *Carex* L. | 0.004 | 0 | 0.002 | RB(DA) |
|  | *Carex* L. | 0.013 | 0.013 | 0.013 | SH |
|  | *Carex* L. 01 | 0.004 | 0 | 0.002 | CH(DA) |
|  | *Carex* L. 01 | 0.009 | 0 | 0.004 | MA(DA) |
|  | *Carex* L. 011 | 0.017 | 0.017 | 0.017 | LR |
|  | *Carex* L. 011 | 0.013 | 0.009 | 0.011 | MV |
|  | *Carex* L. 02 | 0.004 | 0 | 0.002 | CH(DA) |
|  | *Carex* L. 02 | 0.004 | 0 | 0.002 | GF(DA) |
|  | *Carex* L. 02 | 0.009 | 0 | 0.004 | MA(DA) |
|  | *Carex* L. 022 | 0.009 | 0.004 | 0.007 | LR |
|  | *Carex* L. 022 | 0.004 | 0 | 0.002 | MV(DA) |
|  | *Carex* L. 03 | 0.009 | 0.009 | 0.009 | LR |
|  | *Carex* L. 03 | 0.009 | 0.009 | 0.009 | MV |
|  | *Carex* L. 04 | 0.013 | 0.004 | 0.009 | MV |
|  | *Carex* L. 05 | 0.004 | 0 | 0.002 | MV(DA) |
|  | *Carex* L. 06 | 0.026 | 0.017 | 0.022 | WP |
|  | *Carex* L. 07 | 0.009 | 0.009 | 0.009 | WP |
|  | *Carex* L. 08 | 0.004 | 0.009 | 0.007 | WP |
|  | *Carex* L. 09 | 0.009 | 0.013 | 0.011 | WP |
|  | *Carex* L. 11 | 0.013 | 0.004 | 0.009 | WP |
|  | *Carex* L. 12 | 0.004 | 0.004 | 0.004 | WP |
|  | *Carex* L. 13 | 0 | 0.009 | 0.004 | WP(DE) |
|  | *Carex* L. M | 0.004 | 0.009 | 0.007 | R1 |
|  | *Carex* L. M | 0.009 | 0.009 | 0.009 | Z1 |
|  | *Carex* L. N | 0.004 | 0.013 | 0.009 | R1 |
|  | *Carex* L. N | 0.009 | 0.009 | 0.009 | Z1 |
|  | *Carex* L. W | 0.004 | 0 | 0.002 | R1(DA) |
|  | *Carya* Nutt. | 0.009 | 0.022 | 0.015 | AN |
|  | *Carya* Nutt. | 0.004 | 0.009 | 0.007 | CH |
|  | *Carya* Nutt. | 0.009 | 0.009 | 0.009 | CR |
|  | *Carya* Nutt. | 0.013 | 0.013 | 0.013 | GF |
|  | *Carya* Nutt. | 0.004 | 0.009 | 0.007 | GM |
|  | *Carya* Nutt. | 0.004 | 0.031 | 0.017 | MA |
|  | *Carya* Nutt. | 0.004 | 0.017 | 0.011 | MO |
|  | *Carya* Nutt. | 0.009 | 0 | 0.004 | SH(DA) |
|  | *Carya* Nutt. | 0.004 | 0 | 0.002 | WP(DA) |
|  | *Carya* Nutt. 2 | 0 | 0.004 | 0.002 | GF(DE) |
|  | *Conyza* Less. | 0.009 | 0.009 | 0.009 | CA |
|  | *Cornus* L. | 0.004 | 0.004 | 0.004 | MP |
|  | *Crataegus* L. | 0 | 0.004 | 0.002 | CR(DE) |
|  | *Crataegus* L. | 0.009 | 0 | 0.004 | FE(DA) |
|  | *Crataegus* L. | 0 | 0.004 | 0.002 | GF(DE) |
|  | *Crataegus* L. | 0.004 | 0 | 0.002 | R1(DA) |
|  | *Cynoglossum* L. | 0 | 0.004 | 0.002 | CA(DE) |
|  | *Cyperaceae* | 0.013 | 0 | 0.007 | AN(DA) |
|  | DCY | 0 | 0.004 | 0.002 | SH(DE) |
|  | *Desmodium* Desv. | 0.009 | 0.004 | 0.007 | CR |
|  | *Desmodium* Desv. | 0.026 | 0.009 | 0.017 | MA |
|  | *Desmodium* Desv. | 0.004 | 0 | 0.002 | MG(DA) |
|  | *Desmodium* Desv. | 0 | 0.004 | 0.002 | RB(DE) |
|  | *Desmodium* Desv. | 0.004 | 0.004 | 0.004 | SH |
|  | *Dichanthelium* (Hitchc. & Chase) Gould | 0.004 | 0 | 0.002 | SE(DA) |
|  | *Dichanthelium* (Hitchc. & Chase) Gould | 0.017 | 0.017 | 0.017 | GF |
|  | *Dichanthelium* (Hitchc. & Chase) Gould | 0 | 0.004 | 0.002 | GM(DE) |
|  | *Dichanthelium* (Hitchc. & Chase) Gould | 0.017 | 0.009 | 0.013 | MA |
|  | *Dryopteris* Adans. | 0 | 0.004 | 0.002 | WP(DE) |
|  | *Elymus* L. | 0.022 | 0.022 | 0.022 | CH |
|  | Elymus L. | 0.009 | 0 | 0.004 | MA(DA) |
|  | EUAL | 0 | 0.004 | 0.002 | MA(DE) |
|  | *Euonymus* L. | 0.004 | 0 | 0.002 | AN(DA) |
|  | *Euonymus* L. | 0 | 0.004 | 0.002 | GM(DE) |
|  | *Euonymus* L. | 0.004 | 0 | 0.002 | MA(DA) |
|  | *Euonymus* L. | 0.004 | 0.004 | 0.004 | MO |
|  | *Euonymus* L. | 0.004 | 0 | 0.002 | R1(DA) |
|  | *Euonymus* L. | 0.004 | 0 | 0.002 | RB(DA) |
|  | *Eurybia* (Cass.) Cass. | 0.009 | 0.013 | 0.011 | CH |
|  | *Eurybia* (Cass.) Cass. | 0 | 0.009 | 0.004 | MA(DE) |
|  | *Fabaceae* | 0.009 | 0.004 | 0.007 | MA |
|  | *Fragaria* L. | 0.026 | 0.031 | 0.028 | CH |
|  | *Fragaria* L. | 0.004 | 0.004 | 0.004 | CR |
|  | *Fragaria* L. | 0 | 0.013 | 0.007 | MA(DE) |
|  | *Fraxinus* L. | 0.013 | 0.004 | 0.009 | AN |
|  | *Fraxinus* L. | 0.009 | 0.009 | 0.009 | CH |
|  | *Fraxinus* L. | 0.039 | 0.035 | 0.037 | CR |
|  | *Fraxinus* L. | 0.013 | 0.013 | 0.013 | FE |
|  | *Fraxinus* L. | 0.009 | 0.009 | 0.009 | GF |
|  | *Fraxinus* L. | 0 | 0.004 | 0.002 | GM(DE) |
|  | *Fraxinus* L. | 0.052 | 0.039 | 0.046 | MA |
|  | *Fraxinus* L. | 0.013 | 0.004 | 0.009 | MO |
|  | *Fraxinus* L. | 0 | 0.004 | 0.002 | MP(DE) |
|  | *Fraxinus* L. | 0 | 0.004 | 0.002 | R1(DE) |
|  | *Fraxinus* L. | 0.039 | 0.035 | 0.037 | WP |
|  | *Fraxinus* L. 1 | 0 | 0.004 | 0.002 | CH(DE) |
|  | *Fraxinus* L. 2 | 0 | 0.004 | 0.002 | CH(DE) |
|  | *Galium* L. | 0.004 | 0 | 0.002 | AN(DA) |
|  | *Galium* L. | 0.004 | 0 | 0.002 | CH(DA) |
|  | *Galium* L. | 0 | 0.009 | 0.004 | CR(DE) |
|  | *Galium* L. | 0.004 | 0.009 | 0.007 | GF |
|  | *Galium* L. | 0.013 | 0.009 | 0.011 | MA |
|  | *Galium* L. | 0.009 | 0.009 | 0.009 | MP |
|  | *Galium* L. | 0.004 | 0 | 0.002 | SH(DA) |
|  | *Gaylussacia* Kunth | 0 | 0.009 | 0.004 | VM(DE) |
|  | *Geranium* L. | 0 | 0.004 | 0.002 | CR(DE) |
|  | *Geum* L. | 0.009 | 0.035 | 0.022 | AN |
|  | *Geum* L. | 0.017 | 0.022 | 0.020 | CH |
|  | *Geum* L. | 0.013 | 0.026 | 0.020 | CR |
|  | *Geum* L. | 0.004 | 0.004 | 0.004 | SH |
|  | *Geum* L. | 0.004 | 0 | 0.002 | TR(DA) |
|  | *Geum* L. 1 | 0.004 | 0.009 | 0.007 | CH |
|  | *Glyceria* R. Br. | 0.009 | 0.004 | 0.007 | FE |
|  | *Glyceria* R. Br. | 0.009 | 0.013 | 0.011 | WP |
|  | *Helianthus* L. | 0.004 | 0.004 | 0.004 | FE |
|  | *Hieracium* L. | 0 | 0.009 | 0.004 | MA(DE) |
|  | *Hypericum* L. | 0 | 0.009 | 0.004 | MA(DE) |
|  | *Juncus* L. | 0 | 0.004 | 0.002 | SE(DE) |
|  | *Lamiaceae* | 0.004 | 0 | 0.002 | AN(DA) |
|  | *Leersia* Sw. | 0.022 | 0.013 | 0.017 | LR |
|  | *Leersia* Sw. | 0.022 | 0.017 | 0.020 | MV |
|  | *Ligustrum* L. | 0 | 0.004 | 0.002 | WP(DE) |
|  | *Liliaceae* | 0 | 0.004 | 0.002 | CH(DE) |
|  | *Liliaceae* | 0.004 | 0 | 0.002 | MA(DA) |
|  | *Lonicera* L. | 0 | 0.004 | 0.002 | MP(DE) |
|  | *Lonicera* L. | 0.004 | 0 | 0.002 | SH(DA) |
|  | *Maianthemum* F.H. Wigg. | 0 | 0.009 | 0.004 | SH(DE) |
|  | *Malus* Mill. | 0.004 | 0.017 | 0.011 | CH |
|  | *Monarda* L. | 0 | 0.009 | 0.004 | SH(DE) |
|  | *Morus* L. | 0.004 | 0.009 | 0.007 | AN |
|  | *Morus* L. | 0 | 0.009 | 0.004 | MO(DE) |
|  | *Orchidaceae* | 0 | 0.004 | 0.002 | CH(DE) |
|  | *Osmorhiza* Raf. | 0.004 | 0.004 | 0.004 | CH |
|  | *Osmorhiza* Raf. | 0.004 | 0.009 | 0.007 | CR |
|  | *Oxalis* L. | 0.017 | 0 | 0.009 | AN(DA) |
|  | *Oxalis* L. | 0.009 | 0 | 0.004 | CH(DA) |
|  | *Oxalis* L. | 0.039 | 0.017 | 0.028 | CR |
|  | *Oxalis* L. | 0 | 0.013 | 0.007 | GF(DE) |
|  | *Oxalis* L. | 0.004 | 0.004 | 0.004 | MA |
|  | *Oxalis* L. | 0.004 | 0.004 | 0.004 | MO |
|  | P3 | 0.004 | 0 | 0.002 | CH(DA) |
|  | *Panicum* L. | 0 | 0.004 | 0.002 | GF(DE) |
|  | *Panicum* L. | 0.004 | 0 | 0.002 | GM(DA) |
|  | *Panicum* L. | 0.004 | 0.009 | 0.007 | MA |
|  | *Panicum* L. | 0.009 | 0 | 0.004 | SH(DA) |
|  | *Panicum* L. 1 | 0 | 0.004 | 0.002 | GM(DE) |
|  | *Panicum* L. 2 | 0 | 0.004 | 0.002 | GM(DE) |
|  | PAT | 0.004 | 0 | 0.002 | SH(DA) |
|  | *Pinus* L. | 0 | 0.004 | 0.002 | GF(DE) |
|  | *Poa* L. | 0.004 | 0.009 | 0.007 | SH |
|  | *Poa* L. | 0.004 | 0.004 | 0.004 | WP |
|  | *Poaceae* | 0.013 | 0.013 | 0.013 | AN |
|  | *Poaceae* | 0.031 | 0.017 | 0.024 | CH |
|  | *Poaceae* | 0.057 | 0.022 | 0.039 | CH |
|  | *Poaceae* | 0.022 | 0.017 | 0.020 | CR |
|  | *Poaceae* | 0.009 | 0.009 | 0.009 | GF |
|  | *Poaceae* | 0 | 0.009 | 0.004 | GM(DE) |
|  | *Poaceae* | 0 | 0.004 | 0.002 | LR(DE) |
|  | *Poaceae* | 0.013 | 0.009 | 0.011 | MA |
|  | *Poaceae* | 0.004 | 0 | 0.002 | MO(DA) |
|  | *Poaceae* | 0.017 | 0.013 | 0.015 | SH |
|  | *Poaceae* (Native?) | 0.004 | 0 | 0.002 | MP(DA) |
|  | *Poaceae* 01 | 0 | 0.004 | 0.002 | MA(DE) |
|  | *Poaceae* 02 | 0 | 0.004 | 0.002 | MA(DE) |
|  | *Poaceae* 04 | 0.035 | 0.022 | 0.028 | AN |
|  | *Poaceae* 04 | 0 | 0.004 | 0.002 | CH(DE) |
|  | *Poaceae* 04 | 0.004 | 0.004 | 0.004 | CR |
|  | *Poaceae* 05 | 0.009 | 0.004 | 0.007 | AN |
|  | *Poaceae* 05 | 0.004 | 0.004 | 0.004 | CH |
|  | *Poaceae* 05 | 0 | 0.004 | 0.002 | CR(DE) |
|  | *Poaceae* 06 | 0 | 0.004 | 0.002 | AN(DE) |
|  | *Poaceae* 07 | 0.017 | 0.004 | 0.011 | CA |
|  | *Poaceae* 08 | 0 | 0.009 | 0.004 | SE(DE) |
|  | *Poaceae* 09 | 0.004 | 0.004 | 0.004 | FE |
|  | *Poaceae* 10 | 0.004 | 0 | 0.002 | FE(DA) |
|  | *Poaceae* 12 | 0.017 | 0.017 | 0.017 | WP |
|  | *Poaceae* 13 | 0.013 | 0.004 | 0.009 | WP |
|  | *Poaceae* 16 | 0.004 | 0.004 | 0.004 | WP |
|  | *Polygonatum* Mill. | 0 | 0.004 | 0.002 | MP(DE) |
|  | *Polygonum* L. | 0.048 | 0.026 | 0.037 | AN |
|  | *Polygonum* L. | 0.013 | 0.009 | 0.011 | CH |
|  | *Polygonum* L. | 0.035 | 0.044 | 0.039 | CR |
|  | *Polygonum* L. | 0 | 0.004 | 0.002 | GF(DE) |
|  | *Polygonum* L. | 0 | 0.004 | 0.002 | GM(DE) |
|  | *Polygonum* L. | 0.013 | 0.004 | 0.009 | MO |
|  | *Polygonum* L. | 0.004 | 0 | 0.002 | SH(DA) |
|  | POPU | 0.009 | 0.009 | 0.009 | MA |
|  | *Potentilla* L. | 0.004 | 0 | 0.002 | CR(DA) |
|  | *Potentilla* L. | 0.004 | 0 | 0.002 | GF(DA) |
|  | *Potentilla* L. | 0 | 0.004 | 0.002 | GM(DE) |
|  | PPB | 0.004 | 0 | 0.002 | SH(DA) |
|  | *Prenanthes* L. | 0.004 | 0.004 | 0.004 | R1 |
|  | *Prenanthes* L. | 0.017 | 0 | 0.009 | WP(DA) |
|  | *Prenanthes* L. | 0.004 | 0 | 0.002 | Z1(DA) |
|  | *Prunus* L. | 0.004 | 0.009 | 0.007 | AN |
|  | *Prunus* L. | 0.009 | 0 | 0.004 | CH(DA) |
|  | *Prunus* L. | 0.009 | 0 | 0.004 | CR(DA) |
|  | *Prunus* L. | 0.009 | 0 | 0.004 | SH(DA) |
|  | *Quercus* L. | 0.004 | 0.004 | 0.004 | AN |
|  | *Quercus* L. | 0 | 0.009 | 0.004 | CH(DE) |
|  | *Quercus* L. | 0.009 | 0.009 | 0.009 | CR |
|  | *Quercus* L. | 0.022 | 0.035 | 0.028 | GF |
|  | *Quercus* L. | 0 | 0.004 | 0.002 | GM(DE) |
|  | *Quercus* L. | 0 | 0.017 | 0.009 | MA(DE) |
|  | *Quercus* L. | 0.004 | 0 | 0.002 | MO(DA) |
|  | *Quercus* L. | 0 | 0.004 | 0.002 | RB(DE) |
|  | *Quercus* L. 2 | 0.004 | 0 | 0.002 | GF(DA) |
|  | *Ranunculus* L. | 0.013 | 0.009 | 0.011 | AN |
|  | *Ranunculus* L. | 0.009 | 0.013 | 0.011 | CH |
|  | *Ranunculus* L. | 0.004 | 0.013 | 0.009 | CR |
|  | *Ranunculus* L. | 0 | 0.009 | 0.004 | GF(DE) |
|  | *Ranunculus* L. | 0.004 | 0.004 | 0.004 | MA |
|  | *Ranunculus* L. | 0.004 | 0 | 0.002 | SH(DA) |
|  | *Rhododendron* L. | 0.004 | 0 | 0.002 | SH(DA) |
|  | *Rhus* L. | 0 | 0.004 | 0.002 | SH(DE) |
|  | *Rosa* L. | 0.009 | 0.004 | 0.007 | MA |
|  | *Rosa* L. | 0.009 | 0 | 0.004 | WP(DA) |
|  | *Rosaceae* | 0.004 | 0.004 | 0.004 | SH |
|  | *Rubus* L. | 0.004 | 0.009 | 0.007 | AN |
|  | *Rubus* L. | 0.017 | 0.031 | 0.024 | CA |
|  | *Rubus* L. | 0.017 | 0.017 | 0.017 | CH |
|  | *Rubus* L. | 0.031 | 0.044 | 0.037 | CR |
|  | *Rubus* L. | 0.004 | 0.022 | 0.013 | GF |
|  | *Rubus* L. | 0.004 | 0.013 | 0.009 | GM |
|  | *Rubus* L. | 0.004 | 0.004 | 0.004 | MA |
|  | *Rubus* L. | 0 | 0.004 | 0.002 | MO(DE) |
|  | *Rubus* L. | 0.009 | 0.013 | 0.011 | MP |
|  | *Rubus* L. | 0.009 | 0.009 | 0.009 | R1 |
|  | *Rubus* L. | 0.017 | 0.017 | 0.017 | SH |
|  | *Rubus* L. | 0 | 0.009 | 0.004 | WP(DE) |
|  | *Rubus* L. | 0.013 | 0.013 | 0.013 | Z1 |
|  | *Rubus* L. 1 | 0.004 | 0 | 0.002 | CH(DA) |
|  | *Rubus* L. 1 | 0 | 0.004 | 0.002 | GF(DE) |
|  | *Rubus* L. 2 | 0 | 0.004 | 0.002 | GF(DE) |
|  | *Sanicula* L. | 0 | 0.004 | 0.002 | CH(DE) |
|  | *Sanicula* L. | 0.017 | 0.017 | 0.017 | CR |
|  | *Sanicula* L. | 0.004 | 0 | 0.002 | GF(DA) |
|  | *Sanicula* L. | 0.004 | 0 | 0.002 | R1(DA) |
|  | *Sanicula* L. | 0 | 0.004 | 0.002 | SH(DE) |
|  | *Sanicula* L. | 0 | 0.004 | 0.002 | TR(DE) |
|  | *Sanicula* L. | 0 | 0.004 | 0.002 | VJ(DE) |
|  | *Saxifragaceae* | 0.013 | 0.013 | 0.013 | AN |
|  | *Saxifragaceae* | 0.017 | 0.013 | 0.015 | CR |
|  | *Scutellaria* L. | 0.004 | 0.004 | 0.004 | CH |
|  | *Scutellaria* L. | 0.009 | 0.013 | 0.011 | SE |
|  | *Scutellaria* L. | 0.004 | 0 | 0.002 | SH(DA) |
|  | *Scutellaria* L. | 0.004 | 0.004 | 0.004 | Z1 |
|  | *Sedum* L. | 0 | 0.004 | 0.002 | CH(DE) |
|  | *Setaria* P. Beauv. | 0.004 | 0 | 0.002 | CH(DA) |
|  | *Smilax* L. | 0.017 | 0.013 | 0.015 | CR |
|  | *Smilax* L. | 0 | 0.009 | 0.004 | GF(DE) |
|  | *Smilax* L. | 0.009 | 0.013 | 0.011 | MA |
|  | *Smilax* L. | 0.004 | 0 | 0.002 | SH(DA) |
|  | *Solanum* L. | 0.004 | 0 | 0.002 | AN(DA) |
|  | *Solanum* L. | 0.004 | 0 | 0.002 | CH(DA) |
|  | *Solanum* L. | 0.004 | 0 | 0.002 | CR(DA) |
|  | *Solanum* L. | 0.004 | 0 | 0.002 | MA(DA) |
|  | *Solidago* L. | 0.009 | 0 | 0.004 | AN(DA) |
|  | *Solidago* L. | 0 | 0.004 | 0.002 | CA(DE) |
|  | *Solidago* L. | 0.009 | 0.031 | 0.020 | CH |
|  | *Solidago* L. | 0 | 0.004 | 0.002 | CR(DE) |
|  | *Solidago* L. | 0.026 | 0.017 | 0.022 | SE |
|  | *Solidago* L. | 0.004 | 0.009 | 0.007 | MA |
|  | *Solidago* L. | 0.004 | 0.004 | 0.004 | R1 |
|  | *Solidago* L. | 0.013 | 0.017 | 0.015 | SH |
|  | *Solidago* L. 1 | 0.009 | 0.004 | 0.007 | CH |
|  | *Solidago* L. 2 | 0 | 0.004 | 0.002 | AN(DE) |
|  | *Solidago* L. 3 | 0 | 0.004 | 0.002 | AN(DE) |
|  | *Sonchus* L. 4 | 0.004 | 0 | 0.002 | FE(DA) |
|  | SPG | 0 | 0.004 | 0.002 | SH(DE) |
|  | TALSP | 0.004 | 0 | 0.002 | MO(DA) |
|  | *Thalictrum* L. | 0.004 | 0.004 | 0.004 | SH |
|  | *Trillium* L. | 0 | 0.009 | 0.004 | CH(DE) |
|  | *Trillium* L. | 0 | 0.004 | 0.002 | TR(DE) |
|  | *Ulmus* L. | 0.009 | 0.004 | 0.007 | AN |
|  | *Ulmus* L. | 0.017 | 0.004 | 0.011 | CH |
|  | *Ulmus* L. | 0.035 | 0.022 | 0.028 | CR |
|  | *Ulmus* L. | 0.013 | 0.009 | 0.011 | FE |
|  | *Ulmus* L. | 0.004 | 0 | 0.002 | GF(DA) |
|  | *Ulmus* L. | 0 | 0.009 | 0.004 | GM(DE) |
|  | *Ulmus* L. | 0 | 0.004 | 0.002 | MA(DE) |
|  | *Ulmus* L. | 0.004 | 0 | 0.002 | WP(DA) |
|  | UNK | 0.022 | 0.004 | 0.013 | CR |
|  | UNK | 0.004 | 0 | 0.002 | MO(DA) |
|  | UNKB1 | 0.004 | 0 | 0.002 | MP(DA) |
|  | UNKB10 | 0.004 | 0 | 0.002 | MP(DA) |
|  | UNKB2 | 0.013 | 0.004 | 0.009 | MP |
|  | UNKB3 | 0 | 0.004 | 0.002 | MP(DE) |
|  | UNKB4 | 0.004 | 0.004 | 0.004 | MP |
|  | UNKB5 | 0 | 0.004 | 0.002 | MP(DE) |
|  | UNKB6 | 0 | 0.004 | 0.002 | MP(DE) |
|  | UNKB7 | 0.004 | 0 | 0.002 | MP(DA) |
|  | UNKB8 | 0.004 | 0 | 0.002 | MP(DA) |
|  | UNKB9 | 0 | 0.004 | 0.002 | MP(DE) |
|  | UNKBM | 0 | 0.004 | 0.002 | MP(DE) |
|  | UNKF | 0.026 | 0.009 | 0.017 | AN |
|  | UNKF | 0.031 | 0.017 | 0.024 | CH |
|  | UNKF | 0.031 | 0.035 | 0.033 | CR |
|  | UNKF | 0.004 | 0.004 | 0.004 | GF |
|  | UNKF | 0 | 0.004 | 0.002 | GM(DE) |
|  | UNKF | 0.017 | 0.013 | 0.015 | MA |
|  | UNKF | 0.004 | 0.009 | 0.007 | R1 |
|  | UNKF | 0.017 | 0.017 | 0.017 | SH |
|  | UNKF | 0.004 | 0.004 | 0.004 | Z1 |
|  | UNKF1 | 0.004 | 0.004 | 0.004 | MA |
|  | UNKF2 | 0.013 | 0 | 0.007 | AN(DA) |
|  | UNKF2 | 0.004 | 0.009 | 0.007 | MA |
|  | UNKF2 | 0 | 0.004 | 0.002 | R1(DE) |
|  | UNKF3 | 0.004 | 0 | 0.002 | MA(DA) |
|  | UNKF3 | 0 | 0.004 | 0.002 | R1(DE) |
|  | UNKF4 | 0 | 0.004 | 0.002 | R1(DE) |
|  | UNKFC | 0.004 | 0 | 0.002 | CA(DA) |
|  | UNKFC2 | 0.004 | 0 | 0.002 | CA(DA) |
|  | UNKFE | 0.004 | 0 | 0.002 | CH(DA) |
|  | UNKFE | 0 | 0.004 | 0.002 | CR(DE) |
|  | UNKFE | 0 | 0.004 | 0.002 | GM(DE) |
|  | UNKFE | 0 | 0.004 | 0.002 | R1(DE) |
|  | UNKFEC | 0 | 0.009 | 0.004 | CA(DE) |
|  | UNKFED | 0.004 | 0.004 | 0.004 | SE |
|  | UNKFF1 | 0.004 | 0.004 | 0.004 | FE |
|  | UNKFF3 | 0.004 | 0 | 0.002 | FE(DA) |
|  | UNKFF5 | 0.004 | 0 | 0.002 | FE(DA) |
|  | UNKFF6 | 0 | 0.004 | 0.002 | FE(DE) |
|  | UNKFW1 | 0.009 | 0.009 | 0.009 | WP |
|  | UNKFW2 | 0 | 0.004 | 0.002 | WP(DE) |
|  | UNKFW7 | 0.004 | 0.004 | 0.004 | WP |
|  | UNKFW8 | 0.004 | 0.004 | 0.004 | WP |
|  | UNKS | 0.004 | 0.013 | 0.009 | AN |
|  | UNKS | 0.009 | 0 | 0.004 | CH(DA) |
|  | UNKS | 0.013 | 0 | 0.007 | CR(DA) |
|  | UNKS | 0.057 | 0.048 | 0.052 | GF |
|  | UNKS | 0.013 | 0.004 | 0.009 | GM |
|  | UNKS | 0.009 | 0 | 0.004 | MA(DA) |
|  | UNKS | 0.013 | 0.004 | 0.009 | MO |
|  | UNKS | 0.004 | 0 | 0.002 | RB(DA) |
|  | UNKS1 | 0.004 | 0 | 0.002 | GF(DA) |
|  | UNKS1 | 0 | 0.004 | 0.002 | MO(DE) |
|  | UNKS2 | 0.009 | 0 | 0.004 | GF(DA) |
|  | UNKS2 | 0 | 0.004 | 0.002 | MO(DE) |
|  | UNKS3 | 0.004 | 0 | 0.002 | GF(DA) |
|  | UNKSDED | 0.004 | 0.004 | 0.004 | SE |
|  | UNKSED | 0.017 | 0.004 | 0.011 | SE |
|  | UNKSF | 0 | 0.009 | 0.004 | FE(DE) |
|  | UNKV | 0.004 | 0 | 0.002 | R1(DA) |
|  | UNKW | 0.004 | 0 | 0.002 | CH(DA) |
|  | UNKW | 0.009 | 0 | 0.004 | CR(DA) |
|  | UNKW1 | 0.004 | 0.009 | 0.007 | WP |
|  | UNKW3 | 0 | 0.004 | 0.002 | TR(DE) |
|  | UNKW4 | 0.004 | 0 | 0.002 | TR(DA) |
|  | UNKWS | 0.009 | 0.004 | 0.007 | CH |
|  | UNKWS | 0.031 | 0.017 | 0.024 | GF |
|  | UNKWS | 0.004 | 0.013 | 0.009 | GM |
|  | UNKWS | 0.017 | 0.017 | 0.017 | MA |
|  | UNKWS | 0 | 0.004 | 0.002 | RB(DE) |
|  | UNKWS1 | 0.004 | 0 | 0.002 | GF(DA) |
|  | UNKWS2 | 0.013 | 0.004 | 0.009 | GF |
|  | *Vaccinium* L. | 0.004 | 0 | 0.002 | CR(DA) |
|  | *Vaccinium* L. | 0.013 | 0.009 | 0.011 | MA |
|  | *Vaccinium* L. | 0.009 | 0.013 | 0.011 | SH |
|  | *Viburnum* L. | 0 | 0.013 | 0.007 | GF(DE) |
|  | *Viburnum* L. | 0 | 0.004 | 0.002 | GM(DE) |
|  | *Vicia* L. | 0.004 | 0 | 0.002 | SE(DA) |
|  | *Viola* L. | 0.031 | 0.026 | 0.028 | AN |
|  | *Viola* L. | 0.079 | 0.083 | 0.081 | CH |
|  | *Viola* L. | 0.048 | 0.048 | 0.048 | CR |
|  | *Viola* L. | 0.004 | 0 | 0.002 | FE(DA) |
|  | *Viola* L. | 0.017 | 0.013 | 0.015 | FN |
|  | *Viola* L. | 0.026 | 0.026 | 0.026 | GF |
|  | *Viola* L. | 0.022 | 0.022 | 0.022 | GM |
|  | *Viola* L. | 0.004 | 0 | 0.002 | LR(DA) |
|  | *Viola* L. | 0.009 | 0.004 | 0.007 | MA |
|  | *Viola* L. | 0 | 0.009 | 0.004 | MG(DE) |
|  | *Viola* L. | 0.004 | 0 | 0.002 | MO(DA) |
|  | *Viola* L. | 0.013 | 0 | 0.007 | MP(DA) |
|  | *Viola* L. | 0.009 | 0.009 | 0.009 | R1 |
|  | *Viola* L. | 0.017 | 0.022 | 0.020 | SH |
|  | *Viola* L. | 0.009 | 0.009 | 0.009 | TR |
|  | *Viola* L. | 0.013 | 0.004 | 0.009 | VJ |
|  | *Viola* L. | 0 | 0.004 | 0.002 | WP(DE) |
|  | *Viola* L. | 0.004 | 0.009 | 0.007 | Z1 |
|  | *Viola* L. 1 | 0 | 0.009 | 0.004 | GM(DE) |
|  | *Viola* L. 1 | 0.013 | 0.013 | 0.013 | LR |
|  | *Viola* L. 1 | 0.013 | 0.009 | 0.011 | MV |
|  | *Viola* L. 2 | 0 | 0.009 | 0.004 | GM(DE) |
|  | *Viola* L. 2 | 0.004 | 0.004 | 0.004 | LR |
|  | *Viola* L. 2 | 0.013 | 0.004 | 0.009 | MV |
|  | *Viola* L. 3 | 0 | 0.004 | 0.002 | GM(DE) |
|  | *Vitis* L. | 0.022 | 0.026 | 0.024 | AN |
|  | *Vitis* L. | 0.026 | 0.026 | 0.026 | CA |
|  | *Vitis* L. | 0.039 | 0.026 | 0.033 | CH |
|  | *Vitis* L. | 0.044 | 0.035 | 0.039 | CR |
|  | *Vitis* L. | 0.035 | 0.022 | 0.028 | GF |
|  | *Vitis* L. | 0.022 | 0.013 | 0.017 | GM |
|  | *Vitis* L. | 0.057 | 0.048 | 0.052 | MA |
|  | *Vitis* L. | 0.009 | 0.022 | 0.015 | MO |
|  | *Vitis* L. | 0 | 0.004 | 0.002 | MP(DE) |
|  | *Vitis* L. | 0.004 | 0.009 | 0.007 | R1 |
|  | *Vitis* L. | 0.009 | 0.004 | 0.007 | RB |
|  | *Vitis* L. | 0 | 0.004 | 0.002 | VJ(DE) |
|  | *Vitis* L. | 0.004 | 0.004 | 0.004 | WP |
|  | *Vitis* L. | 0.004 | 0.004 | 0.004 | Z1 |
|  | *Vitis* L. 2 | 0 | 0.004 | 0.002 | MO(DE) |
|  | VMI | 0.004 | 0 | 0.002 | SH(DA) |
| ^a^ See Table 1 in main text for additional site information. | |  |  |  |  |
